# Substitutional Vanadium Sulfide Nanodispersed in MoS_2_ Film for Pt‐Scalable Catalyst

**DOI:** 10.1002/advs.202003709

**Published:** 2021-06-03

**Authors:** Frederick Osei‐Tutu Agyapong‐Fordjour, Seok Joon Yun, Hyung‐Jin Kim, Wooseon Choi, Balakrishnan Kirubasankar, Soo Ho Choi, Laud Anim Adofo, Stephen Boandoh, Yong In Kim, Soo Min Kim, Young‐Min Kim, Young Hee Lee, Young‐Kyu Han, Ki Kang Kim

**Affiliations:** ^1^ Department of Energy Science Sungkyunkwan University Suwon 16419 Republic of Korea; ^2^ Department of Energy and Materials Engineering Dongguk University Seoul 04620 Republic of Korea; ^3^ Center for Integrated Nanostructure Physics (CINAP) Institute for Basic Science (IBS) Sungkyunkwan University Suwon 16419 Republic of Korea; ^4^ Department of Chemistry Sookmyung Women's University Seoul 140742 Republic of Korea

**Keywords:** first‐principles calculations, hydrogen evolution, molybdenum disulfide, transition metal dichalcogenides, vanadium disulfide

## Abstract

Among transition metal dichalcogenides (TMdCs) as alternatives for Pt‐based catalysts, metallic‐TMdCs catalysts have highly reactive basal‐plane but are unstable. Meanwhile, chemically stable semiconducting‐TMdCs show limiting catalytic activity due to their inactive basal‐plane. Here, metallic vanadium sulfide (VS*_n_*) nanodispersed in a semiconducting MoS_2_ film (V–MoS_2_) is proposed as an efficient catalyst. During synthesis, vanadium atoms are substituted into hexagonal monolayer MoS_2_ to form randomly distributed VS*_n_* units. The V–MoS_2_ film on a Cu electrode exhibits Pt‐scalable catalytic performance; current density of 1000 mA cm^−2^ at 0.6 V and overpotential of −0.08 V at a current density of 10 mA cm^−2^ with excellent cycle stability for hydrogen‐evolution‐reaction (HER). The high intrinsic HER performance of V–MoS_2_ is explained by the efficient electron transfer from the Cu electrode to chalcogen vacancies near vanadium sites with optimal Gibbs free energy (−0.02 eV). This study provides insight into ways to engineer TMdCs at the atomic‐level to boost intrinsic catalytic activity for hydrogen evolution.

## Graphical abstract

The nanodispersed vanadium sulfide (VS*_n_*) in the MoS_2_ lattice is key for efficient hydrogen evolution by increasing the electrical conductivity and basal plane activity of V–MoS_2_ catalyst. The engineering advantage is that the stability of VS_2_ is ensured by the embedded VS*_n_* in MoS_2_ and the copper substrate tunes the Gibbs free energy and facilitates electron injection to active sites.

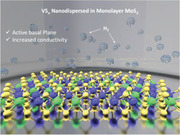

One renewable energy resource is water electrolysis for hydrogen production. Noble metals such as Pt and Pd as catalysts for water electrolysis are inevitably utilized for efficient hydrogen evolution,^[^ ^1^, ^2^, ^3^ ^]^ which have been limited for industry application owing to scarcity and high cost. Therefore, it is highly desired to develop efficient noble‐metal free catalyst. Among a variety of noble‐metal free catalysts, transition metal dichalcogenide (TMdC)‐layered material has been proposed. Metallic TMdCs such as VS_2_,^[^ ^4^ ^]^ VSe_2_,^[^ ^5^ ^]^ and NbS_2_^[^ ^6^ ^]^ demonstrate great potential for hydrogen‐evolution‐reaction (HER) performance owing to their active basal plane and high electrical conductivity but are often not stable in air,^[^ ^7^, ^8^, ^9^ ^]^ and most importantly in HER environment,^[^ ^5^, ^10^ ^]^ which is a primary concern for industrial targets. Meanwhile, semiconducting TMdCs such as MoS_2_ and WS_2_ in monolayer form^[^ ^11^, ^12^, ^13^ ^]^ are stable in air but are inactive in the basal plane and have poor electrical conductivity. Additionally, monolayer TMdCs allow for short electron injection paths from the electrode to active sites to promote efficient HER performance, unlike the bulk materials, which offers limited HER kinetics because of charge lagging to reach active surface sites.^[^ ^14^, ^15^ ^]^

Several approaches with semiconducting TMdCs have been investigated to resolve the basal‐plane inactivity including chalcogen vacancies, phase boundaries, and heterostructures.^[^ ^16^, ^17^, ^18^ ^]^ Although the hydrogen adsorption Gibbs free energy (∆*G*_H*_) approaches nearly zero in chalcogen vacancies and heterostructures, electrical conductivity remains poor, leaving sluggish HER kinetics.^[^ ^14^, ^15^, ^18^ ^]^ Heterophase boundaries are highly reactive in terms of HER but the necessary phase‐boundary line density is rarely encountered.^[^ ^17^ ^]^ Structural transformation from the semiconducting 2H‐phase to metallic 1T phase renders these materials reactive with respect to HER performance but unfavorable owing to the thermodynamically unstable 1T phase.^[^ ^19^ ^]^ Recently, the substitution of metal and chalcogen atoms in bulk s‐TMdCs prepared by hydrothermal method improved the HER performance, but the complicated structures make it difficult to understand the underlying HER mechanism.^[^ ^20^, ^21^, ^22^ ^]^ Our research target is to take advantage of the highly conductive basal plane of monolayer TMdC film in large‐area for efficient HER performance, yet to obtain a material with high stability. Here, we construct a one‐step chemical vapor deposition (CVD) process to directly synthesize metallic vanadium sulfide (VS*_n_*) units nanodispersed in semiconducting monolayer MoS_2_ film (V–MoS_2_) and further demonstrate superb HER performance via overpotential, hydrogen turnover frequency (TOF), cycle test, and Gibbs free energy that are comparable to those of Pt catalyst.

**Figure** **1a** depicts the ball‐and‐stick model for randomly dispersed VS*_n_* unit in the active basal plane of a hexagonal MoS_2_ monolayer. Most importantly, the chalcogen vacancy is created near the vanadium site, which is defined as a VS*_n_* unit. This unit plays an important role as active sites in the basal plane to promote both efficient charge transfer and hydrogen adsorption. The nanodispersed VS*_n_* units in monolayer MoS_2_ film was synthesized by one‐step CVD process (see Experimental Section and Figure S1, Supporting Information). Atomic structures and the homogeneity of V distribution in V–MoS_2_ at 9.3 at% V concentration are provided in an annular dark field scanning transmission electron microscopy (ADF‐STEM) image (Figure 1b and Figures S2 and S3, Supporting Information). We note that the bright contrast features are frequently observed on top of the samples in the ADF‐STEM images. This is confirmed from the poly(methyl methacrylate) (PMMA) residues introduced for transfer of the samples, but not from the presence of V clusters (Figures S4–S6, Supporting Information). The *d*‐spacing between S–S in the VS*_n_* unit, which is confirmed by observing the ($10\bar{1}0)$ plane from the electron diffraction pattern shown in the inset, is 0.27 nm, similar to that of pristine 2H–MoS_2_ (0.27 nm).^[^ ^23^ ^]^ The real and simulated ADF‐STEM images (the square region in Figure 1b) show the atomic configuration of the VS*_n_* unit region with an S vacancy in V–MoS_2_ (Figure 1c). Furthermore, the intensity profile clearly distinguishes among Mo, S, and V atoms and the S vacancy next to the V atom (V‐vac_s_) (Figure 1d and inset: top view of the atomic configuration). These results indicate that the V atoms are well‐substituted into Mo sites in the 2H–MoS_2_ lattice with negligible strains. The formation of VS*_n_* units was readily confirmed by newly emerged peak in Raman spectra and reduced photoluminescence intensity due to the enhanced metallic character (Figure S1, Supporting Information). The presence of V atoms in the as‐grown V–MoS_2_ film was additionally confirmed by energy dispersive X‐ray spectroscopy and X‐ray photoelectron spectroscopy (Figures S7–S9, Supporting Information).

**Figure 1:**
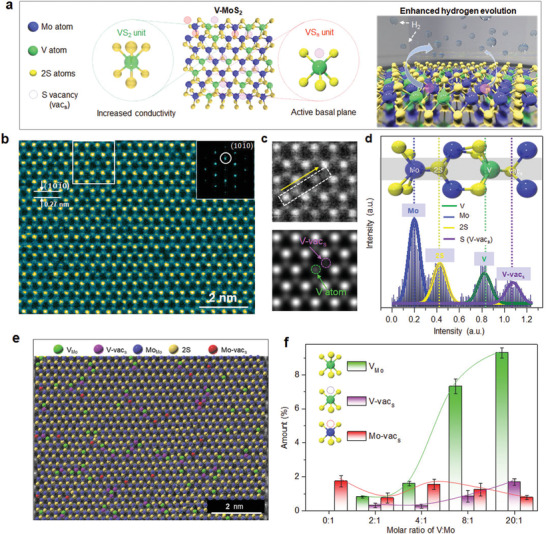
Atomic structure of monolayer V–MoS_2_. a) Schematic of V–MoS_2_ with VS_2_ and VS*_n_* units and hydrogen evolution on V–MoS_2_ via basal‐plane activation. b) ADF‐STEM image at 9.3% V concentration, indicating a *d*‐spacing of 0.27 nm for 2H–MoS_2_ and the corresponding electron‐diffraction‐pattern of ($10\bar{1}0$) plane in the inset. c) STEM image of white square region in (b) and simulated image and d) the corresponding intensity profile. e) False‐colored ADF‐STEM image of monolayer V–MoS_2_ with Mo‐substituted V atom (V_Mo_), sulfur‐vacancy next to V atom (V‐vac_s_), Mo atom (Mo_Mo_), two S atoms (2S), and sulfur‐vacancy next to Mo atom (Mo‐vac_s_). f) Atomic % distribution of V_Mo_, V‐vac_s_, and Mo‐vac_s_ as a function of molar ratio of V to Mo precursor. Statistical analysis data were obtained from false‐colored ADF‐STEM images.

We carefully analyzed the atomic configurations of V at the Mo site (V_Mo_), V‐vac_s_, Mo at the Mo site (Mo_Mo_), the S vacancy next to the Mo atom (Mo‐vac_s_), and the S dimer (2S) (Figure 1e and Figure S10, Supporting Information). Interestingly, the amount of V‐vac_s_ is proportional to the V_Mo_ concentration, the molar ratio of V to the Mo precursor (Figure 1f and Figures S11–S15, Supporting Information for more details), with a similar trend to that of the longitudinal acoustic mode phonon in Raman spectra originating from impurity levels^[^ ^24^ ^]^ (Figure S16, Supporting Information). The coverage of monolayer region reaches up to 95% (5% multilayer region) in V_(9.3%)_–MoS_2_ (Figures S17 and S18, Supporting Information). Meanwhile, the amount of Mo‐vac_s_ in V–MoS_2_ is stochastically populated up to 2.1%, regardless of V_Mo_ amount (Figure 1f). The higher V‐vac_s_ amount of ≈22.0% per V atom than Mo‐Vac_s_ amount of 1.2% per Mo atom is congruent with our theoretical calculations, where the formation energy of V‐vac_s_ is 0.22 eV more stable than that of Mo‐vac_s_ (Table S1, Supporting Information). The ample V_Mo_ and V‐vac_s_ sites are the key ingredient for efficient electron transfer and hydrogen adsorption, which will be discussed later.

**Figure** **2a** shows the polarization curves in the linear sweep voltammetry plot with varying V concentration and substrate (see Experimental Section for a detailed description of the measurements). Ni and Cu electrodes were chosen for their earth abundance and relatively high electrical conductivities in comparison with conventional graphite (Gr). The polarization curve of V–MoS_2_ with the Gr substrate shifts to the Pt curve with increasing V concentration, surpassing that of pristine MoS_2_. The overpotential with the Ni substrate is further reduced relative to that with the Gr substrate (Figure 2b). It is remarkable to see that with the Cu substrate, V–MoS_2_ shows a similar overpotential in low current density region to that of Pt.

**Figure 2:**
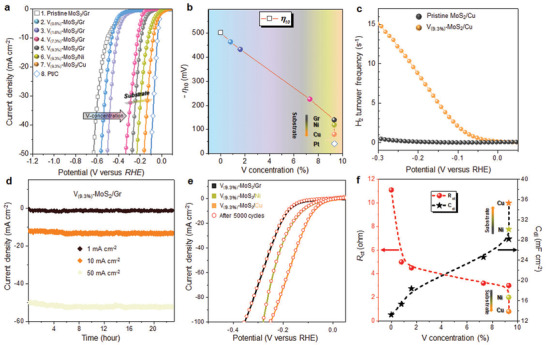
HER activity of V–MoS_2_ in terms of V concentration and substrate. a) Polarization curves for pristine MoS_2_ on graphite (Gr) substrate, V_(0.8%)_–MoS_2_/Gr, V_(1.6%)_–MoS_2_/Gr, V_(7.3%)_–MoS_2_/Gr, V_(9.3%)_–MoS_2_/Gr, V_(9.3%)_–MoS_2_/Ni, V_(9.3%)_–MoS_2_/Cu, and Pt measured in N_2_ saturated 0.5 m H_2_SO_4_ electrolyte at 25 °C at a scan rate of 5 mV s^−1^. b) Overpotential at 10 mA cm^−2^ (*η*_10_) of V–MoS_2_ samples. c) Turnover frequency of V_(9.3%)_–MoS_2_/Cu compared to pristine MoS_2_. d) Chronoamperometric curves of V_(9.3%)_–MoS_2_/Gr under static current densities of 1, 10, and 50 mA cm^−2^ for 24 h. e) Accelerated degradation test of V_(9.3%)_–MoS_2_/Gr, Cu, and Ni substrates after 5000 CV cycles. f) Charge transfer resistance (*R*_ct_) and double layer capacitance (*C*_dl_) of V–MoS_2_ samples as a function of V concentration. The substrate dependence on Gr, Ni, and Cu at V_(9.3%)_–MoS_2_.

The overpotential for 10 mA cm^−2^ (*ɳ*_10_) for assessing catalytic efficiency are extracted from linear sweep voltammetry plot.^[^ ^25^ ^]^ The overpotential gradually decreases to −0.19 V in V_(9.3%)_–MoS_2_ with the Gr substrate. (Figure 2b). The *ɳ*_10_ is further improved by using a different substrate. In particular, V_(9.3%)_–MoS_2_ with the Cu substrate demonstrates the lowest *ɳ*_10_ (−0.08 V) and a hydrogen TOF of 0.3 s^−1^ at 0 V (Figure 2c), comparable to those of Pt^[^ ^26^ ^]^ (*ɳ*_10_ of −0.04 V and TOF of 0.7 s^−1^). Furthermore, the current density reaches to 1000 mA cm^−2^ at 0.6 V, which is viable in industrial requirements (Figure S19, Supporting Information). The outstanding onset potential and *ɳ*_10_ of V_(9.3%)_–MoS_2_ are further compared with those of other 2D materials (Figure S20, Supporting Information).

To evaluate the stability in HER environment, we perform chronoamperometry measurements and an accelerated degradation test. The V_(9.3%)_–MoS_2_ sample clearly demonstrates no significant drop in the current density at different current densities of 1, 10, and 50 mA cm^−2^ for 24 h (Figure 2d). Additionally, the polarization curves are fully superimposed after 5000 cycles, regardless of the substrate used (Figure 2e). Also, the chemical shifts, the change of morphology, and physical structure before and after cycling were negligible (X‐ray photoelectron spectroscopy, scanning electron microscopy (SEM), and Raman analysis in Figure S21, Supporting Information). This superb stability is crucial to meet industrial target and is well‐contrasted with that of pure metallic VS_2_.^[^ ^4^ ^]^

To investigate the origin of the charge transfer kinetics and catalytically active sites in V–MoS_2_, we measured the charge‐transfer resistance (*R*_ct_) and double‐layer capacitance (*C*_dl_) by using impedance spectroscopy and cyclic voltammetry, respectively (see Experimental Section and Figures S22–S25, Supporting Information). The *R*_ct_ rapidly drops at the minute concentration of 0.8 at% V from that of pristine MoS_2_ and gradually reduces to saturate at a concentration of 9.3 at% V (Figure 2f). Such a drastic reduction in *R*_ct_ is ascribed to the degenerate metallic VS_2_ of the high concentration of 9.3 at% V in the semiconducting MoS_2_ lattice.^[^ ^27^ ^]^ The *R*_ct_ for V_(9.3%)_–MoS_2_/Cu is reduced to 0.8 Ω, which is the lowest *R*_ct_ value ever recorded for a 2D monolayer TMdC electrocatalyst to date, and even lower than that of Pt (2.2 Ω) (Figure S22, Supporting Information). The electrochemically active surface area (EASA) extracted from the *C*_dl_^[^ ^28^ ^]^ is nearly negligible for pristine MoS_2_ compared to that of Gr (Figure S25 and Table S2, Supporting Information). This value is gradually elevated with the V concentration, reaching to 28.2 mF cm^−2^ for V_(9.3%)_–MoS_2_, twice as high as that of pristine MoS_2_ (13.2 mF cm^−2^). The EASA with the Cu substrate in V_(9.3%)_–MoS_2_ is further improved from the Gr substrate, ensuring the importance of substrate for industrial applications. Furthermore, EASA normalized polarization curves demonstrate outstanding HER performance of V–MoS_2_/Cu as compared to other materials (Figure S26 and Table S3, Supporting Information). We note that HER characteristics are highly reproducible although the multilayer portions slightly vary with different batches of samples (Figures S27−S30 and Table S4, Supporting Information).

Since the catalytically active sites are directly related to EASA, we elucidate the underlying mechanism on active sites in V–MoS_2_ by performing density functional theory (DFT) calculations. Here, we focus on two main aspects: the Gibbs free energy (∆*G*_H*_) with substrates and density of states (DOS) near the Fermi level associated with active sites. Typical chalcogen vacancies on pure MoS_2_ and V–MoS_2_ and various substrates including Gr (0002), Cu (111), and Ni (111) are schematically drawn in **Figure** **3a**. The Gibbs free energy at chalcogen vacancy next to Mo sites (Mo‐vac_s_) shows relatively close to the thermoneutral point, although its dependence on the substrate varies slightly (Figure 3b). It is remarkable to reveal the best Gibbs free energy at chalcogen vacancy next to V site (V‐vac_s_) on the Cu substrate is −0.02 eV, nearly ideal 0 eV, while the other substrates (Gr and Ni) are far deviated from the thermoneutral point. The volcano plot is summarized with various materials in the literature (Figure 3c and Figures S31–S33, Supporting Information). While the Gibbs free energy of Mo‐vac_s_ is similar to that of V‐vac_s_ with Cu substrate, the exchange current density of V‐vac_s_ is superior to that of Mo‐vac_s_ (Table S5, Supporting Information). A summary of catalytic parameters of V–MoS_2_/Cu in comparison with those of Pt and other TMdCs catalysts are provided in **Table** **1**.

**Figure 3:**
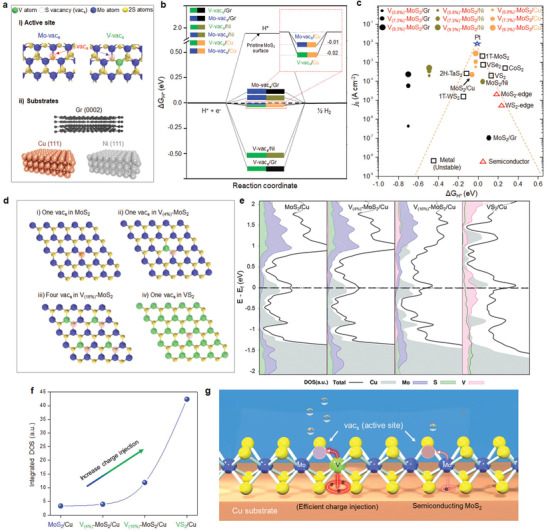
Gibbs free energy for hydrogen adsorption and density of states near the Fermi level of V–MoS_2_ from DFT calculations. a) Schematic of active sites of sulfur vacancy in pristine MoS_2_ and V–MoS_2_ with substrates of Gr (0002), Cu (111), and Ni (111). b) Gibbs free energy diagram of V–MoS_2_ on Ni, Cu, and Gr substrates. c) Volcano plot of other TMDs and our materials (V–MoS_2_ on Cu, Ni, and Gr) with Gibbs free energy (∆*G*_H*_) and exchanged current density (*j*_0_).^[^ ^26^, ^29^, ^30^, ^31^, ^32^, ^33^, ^34^ ^]^ d) Supercell models for MoS_2_, V–MoS_2_ (low V and high V concentration), and VS_2_ with sulfur vacancy. e) Projected density of states for Cu, Mo, S, and V atoms. f) Integrated density of states with V–MoS_2_/Cu. g) Schematic of HER process in V–MoS_2_/Cu catalyst.

**Table 1:** The comparison of catalytic parameters of V–MoS_2_/Cu with Pt and other TMdCs. The comparison of Gibbs free energy for adsorbed hydrogen (∆*G*_H*_), overpotential at 10 (*ɳ*_10_) mA cm^−2^ current density, charge transfer resistance (*R*_ct_), and the turnover frequency (TOF) at 0 V of V–MoS_2_, Pt, and other TMdCs electrocatalysts

Catalyst	∆*G*_H*_ [eV]	*η*_10_ [V]	*R*_ct_ [Ω]	TOF @ 0 V [s^−1^]	Reference
Pt	≈0.00	−0.04	2.2	0.70	Our work
V–MoS_2_/Cu	−0.02	−0.08	0.80	0.30	Our work
Nb_1.35_S_2_	0.11	−0.15	7.40	0.20	^[^ ^6^ ^]^
1T WS_2_	0.28	−0.25	N/A	0.04	^[^ ^31^ ^]^
VS_2_	−0.03	−0.08	27	N/A	^[^ ^4^ ^]^
1T MoS_2_	0.29	−0.22	211	N/A	^[^ ^34^ ^]^

The charge‐transfer to the active site is directly related to the DOS near the Fermi level.^[^ ^6^, ^35^ ^]^ We consider the S vacancies with pristine MoS_2_, V–MoS_2_ with two V concentrations, and VS_2_ on Cu substrate (Figure 3d) and calculate projected DOS (PDOS) of individual atoms in (5 × 5) unit cell (see Experimental Section). The PDOS of MoS_2_ with a single S vacancy (Figure 3e) is developed near the Fermi level (*E* = *E*_F_) and is further developed with additional V site in V_(4%)_–MoS_2_/Cu. The bandgap is still preserved at this V concentration. In addition to enhanced DOS at V_(16%)_–MoS_2_/Cu, the bands are highly degenerate. The bandgap is closed in metallic VS_2_/Cu and therefore the stability of material is no longer guaranteed. The integrated DOS near the Fermi level are elevated with increasing V concentrations and promotes the electron injection to active sites. The higher the V concentration, the better the exchange current density, but limited by material stability like pure VS_2_ (Figure 3f).

The nanodispersed VS*_n_* in the semiconducting MoS_2_ lattice in our study is certainly advantageous in several respects (Figure 3g). The content of chalcogen vacancies in V–MoS_2_ is ≈1.0 × 10^14^ cm^−2^ at 9.3 at% V concentration, which is about twice the amount of S vacancies in pristine MoS_2_.^[^ ^35^ ^]^ Consequently, the exchange current density meets the industrial target. An additional advantage is that the substrate is highly susceptible to proximate monolayer V–MoS_2_, which can be used to engineer Gibbs free energy and electron injection. In particular, the Cu substrate is not only useful to improve the exchange current density but also to tune the Gibbs free energy to the ideal 0 eV, facilitating efficient electron transfer from the Cu substrate to active sites. Another engineering point is preserving the material stability. In our case, the unstable VS_2_ metal is stabilized by introducing nanodispersed VS*_n_* in the semiconducting MoS_2_ lattice. At this stage, raising the V concentration beyond 10% V is limited from a synthesis point of view due to incomplete formation of fully covered V–MoS_2_ film (Figure S34, Supporting Information). This is an open question to increase further the V concentration in order to improve catalyst efficiency, while ensuring stability. The exchange current density can be further improved by introducing 3D scaffolds such as wrinkles or a porous network. Our strategy provides insight into ways to engineer a single‐atom catalyst at the atomic level with 2D materials and furthermore facilitates the design of target‐specific characteristics for application to a variety of electrocatalysts, photocatalysts, and electronic devices.

## Experimental Section

### Growth of MoS_2_ and V–MoS_2_

V–MoS_2_ was synthesized using a one‐step CVD method. Liquid metal precursors were prepared by mixing two aqueous solutions containing Mo and V precursors, respectively (0.05 m sodium molybdate dihydrate [Na_2_MoO_4_·2H_2_O], Sigma‐Aldrich, 331058 and 0.05 m sodium orthovanadate dihydrate [Na_3_VO_4_·2H_2_O], Sigma‐Aldrich, S6508). These solutions were mixed in the given ratios to control the concentration of V in MoS_2_ lattice. The mixed solution was spin‐coated onto a SiO_2_/Si wafer at 2500 revolution‐per‐minute for 1 min. For the growth of the V–MoS_2_ film by CVD, the temperature was elevated to 850 °C under Ar atmosphere at a flow rate of 350 sccm and then dimethyl disulfide as a source of S and H_2_ at flow rates of 3 and 5 sccm, respectively, were introduced for 10 min. After the growth of the V–MoS_2_ film, the temperature was naturally cooled under Ar and H_2_ atmosphere without changing the flow rate. The pristine MoS_2_ film was synthesized by using the same CVD procedure without adding the V precursor.

### Transmission Electron Microscopy and Specimen Preparation

Atomic‐resolution ADF‐STEM images of the samples were acquired using a probe aberration‐corrected STEM (JEM‐ARM200CF, Jeol Ltd.) operating at 80 keV. The detector angle range for ADF imaging was 45–180 mrad and the convergence semi‐angle of the probe was 23 mrad. To avoid electron beam damage, the acquisition time of STEM image was conducted within 10 s (Figure S35, Supporting Information). The multislice method was used for ADF‐STEM image simulations, which was implemented in an open software, QSTEM software package^[^ ^36^ ^]^ and the atomic quantifications from the ADFSTEM images were performed with commercial software qHAADF (HREM Research Ltd.). The TEM sample was prepared by the conventional transfer method with a PMMA C4 (MicroChem) supported layer.^[^ ^18^ ^]^ After transferring the V–MoS_2_ film onto the TEM grids (PELCO, 200 mesh, copper, 1.2 µm holes), the PMMA layer was removed by acetone. To avoid polymerization residuals during STEM imaging, the V–MoS_2_ on the TEM grid was annealed at 300 °C for 3 h under the forming gas atmosphere prior to TEM analysis.

### Surface Morphology and Chemical State Analysis

The surface morphology of the as‐grown V–MoS_2_ film was examined by optical microscopy (Nikon LV‐IM, Nikon) and SEM (JSM‐7100F, JEOL). X‐ray photoemission spectroscopy (K‐Alpha, THERMO FISHER) was employed to characterize the elemental composition of V‐doped MoS_2_. Confocal Raman and PL measurements were conducted using a Nanobase system (XperRam 100, Nanobase) with an excitation energy of 2.32 eV.

### Electrode Fabrication

The as‐grown V–MoS_2_ films (1 × 1 cm^2^) were transferred onto the working electrode (graphite sheet) using a PMMA‐supported wet‐transfer method.^[^ ^18^ ^]^ The PMMA was removed in hot acetone for 10 min to obtain a V–MoS_2_/graphite sheet with a 1 × 1 cm^2^ active geometric surface area. The procedure was repeated for transfer onto other electrodes (Cu and Ni).

### Electrochemical Measurements

All electrochemical measurements were conducted on a ZIVE SP2 (ZIVE Lab, Korea) electrochemical workstation within a three‐electrode cell in 0.5 m H_2_SO_4_ at room temperature. A graphite substrate, saturated calomel electrode (SCE), and graphite rod were used as the working, reference, and counter electrodes, respectively. The electrolyte was de‐aerated by purging with N_2_ for 30 min prior to conducting the electrochemical experiment. Purging was maintained throughout the experiment. The catalytic behavior was characterized using LSV, EIS, cyclic voltammetry (CV), and chronoamperometry measurements. The LSV was measured in the range of 0 to −0.8 V (vs RHE) at a scan rate of 5 mV s^−1^. EIS was measured from 1000 kHz to 10 mHz at an amplitude of 10 mV s^−1^ and a constant potential of −0.3 V. A simple Randles circuit was applied to fit the EIS data using the software Zview. The *C*_dl_ was measured by CV between 0.1 and 0.2 V (vs RHE) at scan rates of 5, 10, 20, 30, 40, 50, 60, 80, and 100 mV s^−1^. The stability and durability were studied using chronoamperometry at −0.27, −0.14, and −0.07 V and CV between −0.3 and 0.1 V (vs RHE) at a scanning rate of 100 mV s^−1^, respectively. The potential calibration of the reference electrode (SCE) was performed in high purity hydrogen (H_2_) 0.5 m H_2_SO_4_ solution using Pt foil as working electrode.^[^ ^37^ ^]^ CV measurement was carried out at 1 mV s^−1^ scan rate. The average of the two potentials which cross the current at zero was taken as a thermodynamic potential for hydrogen electrode reaction. Therefore, all potentials were converted to RHE using the equation:

(1) $$E_{\mathrm{RHE}}=E_{\mathrm{SCE}}+E_{\mathrm{SCE}}^{0}$$

where *E*_RHE_ was the converted potential value versus RHE, *E*_SCE_ was the potential reading from the potentiostat, and $E_{\mathrm{SCE}}^{0}$ was the experimentally determined standard electrode potential of SCE (0.246 V). A resistance test was conducted prior to measurements, and IR compensation was applied using ZIVE SP2 workstation software. The Ohmic drop was corrected using the current interrupt method, and all potentials were IR‐corrected with a compensation level of 90%.

### Turnover‐Frequency Calculation

According to a previous reference,^[^ ^6^ ^]^ the hydrogen TOF could be calculated using the formula:

(2) $$\mathrm{TOF}\left(s^{-1}\right)=\left(\frac{J(Acm^{-2})}{n\times N\times \mathrm{relative}\;\mathrm{EASA}\;\times \left(1.602\times 10^{-19}C\right)}\right)$$

where *n*, *N*, and relative EASA were the number of electrons required to evolve one mole of hydrogen molecule, the density of active sites, and the EASA of V–MoS_2_ with respect to the Cu substrate, respectively.

(3) $$\mathrm{Relative}\;\mathrm{EASA}=\frac{35.3\;\mathrm{mF}\;cm^{-2}}{13.8\;\mathrm{mF}\;cm^{-2}\;}=2.73$$

The lattice parameters *a* = 3.192 Å and *c* = 13.378 Å were used for V–MoS_2_, therefore the surface area of the unit cell was 5.78 × 10^−16^ cm^2^. Thus, the number of active sites was estimated to be ≈1.73 × 10^15^ cm^−2^ presuming that the entire basal plane was catalytically active. Therefore, the geometric density of active sites of V–MoS_2_ was

(4) $$1.73\times 10^{15}cm^{-2}\times 2.73=4.72\times 10^{15}cm^{-2}$$

This was overestimated due to highest active sites at the basal plane. The TOF could be larger in real system. To estimate the TOF at the exchange current density, the TOF was extrapolated linearly from the TOF curve at 0 V.

### Computational Methods

The spin‐polarized DFT calculations were conducted using the Vienna ab initio simulation package.^[^ ^38^ ^]^ The authors employed the revised Perdew–Burke–Ernzerhof type exchange and correlation functional^[^ ^39^ ^]^ combined with the introduction of vdW‐DF^[^ ^40^ ^]^ for the non‐local correlation part, to accurately account for the dispersion interactions.^[^ ^41^ ^]^ The projector augmented wave method was used for ion interaction. The Brillouin zone was sampled using a 3 × 3 × 1 k‐point mesh, while the electronic states were smeared using the Methfessel–Paxton scheme with a broadening width of 0.1 eV. A 6 × 6 × 1 k‐point mesh was used for the DOS calculation. The electronic wave functions were expanded in a plane wave basis with a cutoff energy of 500 eV and the atomic relaxation was continued until the Hellmann–Feynman forces acting on the atoms were less than 0.02 eV Å^−1^. 2D structures were modeled using a 5 × 5 supercell of MoS_2_, and Cu and Ni substrates were modeled by employing slabs consisting of four atomic layers with 2D structures added. A 4 × 8 supercell of MoS_2_ on top of a 5 × 10 supercell of the graphene model was used for the MoS_2_/graphene structure. In each system, a vacuum layer of 16 Å was added onto the MoS_2_ surface to eliminate possible interlayer interactions. The free energy of H adsorption, Δ*G*_H*_, was defined as Δ*G*_H*_ = Δ*E*_H_ + Δ*E*_ZPE_ − *T*Δ*S*_H_, where Δ*E*_H_ was the H adsorption energy, Δ*E*_ZPE_ was the zero‐point energy difference, *T* was room temperature, Δ*S*_H_ was the difference in entropy, and Δ*E*_ZPE_ − *T*Δ*S*_H_ was 0.24 eV.^[^ ^42^ ^]^ Δ*E*_H_ was defined as Δ*E*_H_ = *E*(H* + surface) − *E*(surface) − 1/2*E*(H_2_), where *E*(H* + surface), *E*(surface), and *E*(H_2_) represented the total energies of the H adsorbed surface, pristine surface, and H_2_ molecule in the gas phase, respectively.

## Conflict of Interest

The authors declare no conflict of interest.

## Supporting information

Supporting Information

## Data Availability

The data that support the findings of this study are available from the corresponding author upon reasonable request.

## References

[1] V. R. Stamenkovic, B. S. Mun, M. Arenz, K. J. J. Mayrhofer, C. A. Lucas, G. Wang, P. N. Ross, N. M. Markovic, Nat. Mater. 2007, 6, 241. 17310139 10.1038/nmat1840

[2] E. Casado‐Rivera, D. J. Volpe, L. Alden, C. Lind, C. Downie, T. Vazquez‐Alvarez, A. C. D. Angelo, F. J. DiSalvo, H. D. Abruna, J. Am. Chem. Soc. 2004, 126, 4043. 15038758 10.1021/ja038497a

[3] K. Qi, S. Yu, Q. Wang, W. Zhang, J. Fan, W. Zheng, X. Cui, J. Mater. Chem. A 2016, 4, 4025.

[4] Y. Qu, M. Shao, Y. Shao, M. Yang, J. Xu, C. T. Kwok, X. Shi, Z. Lu, H. Pan, J. Mater. Chem. A 2017, 5, 15080.

[5] Q. Zhu, M. Shao, S. H. Yu, X. Wang, Z. Tang, B. Chen, Z. Lu, D. Chua, H. Pan, ACS Appl. Energy Mater. 2019, 2, 644.

[6] J. Yang, A. R. Mohmad, Y. Wang, R. Fullon, X. Song, F. Zhao, I. Bozkurt, M. Augustin, E. J. G. Santos, H. S. Shin, W. Zhang, D. Voiry, H. Y. Jeong, M. Chhowalla, Nat. Mater. 2019, 18, 1309. 31451781 10.1038/s41563-019-0463-8

[7] S. Shi, Z. Sun, Y. H. Hu, J. Mater. Chem. A 2018, 6, 23932.

[8] W. Yu, J. Li, T. S. Herng, Z. Wang, X. Zhao, X. Chi, W. Fu, I. Abdelwahab, J. Zhou, J. Dan, Z. Chen, Z. Chen, Z. Li, J. Lu, S. J. Pennycook, Y. P. Feng, K. P. Loh, Adv. Mater. 2019, 31, 1903779. 10.1002/adma.201903779 31423650

[9] R. Yan, G. khalsa, B. T. Schaefer, A. Jarjour, S. Rouvimov, K. C. Nowack, H. G. Xing, D. Jena, Appl. Phys. Express 2019, 12, 023008.

[10] H. Liang, H. Shi, D. Zhang, F. Ming, R. Wang, J. Zhuo, Z. Wang, Chem. Mater. 2016, 28, 5587.

[11] S. J. Yun, S. H. Chae, H. Kim, J. C. Park, J. H. Park, G. H. Han, J. S. Lee, S. M. Kim, J. Seok, M. S. Jeong, K. K. Kim, Y. H. Lee, ACS Nano 2015, 9, 5510. 25873415 10.1021/acsnano.5b01529

[12] S. Boandoh, S. H. Choi, J. H. Park, S. Y. Park, S. Bang, M. S. Jeong, J. S. Lee, H. J. Kim, J. Y. Choi, S. M. Kim, K. K. Kim, Small 2017, 13, 1701306. 10.1002/smll.201701306 28834243

[13] S. H. Choi, S. Boandoh, Y. H. Lee, J. S. Lee, J. H. Park, S. M. Kim, W. Yang, K. K. Kim, ACS Appl. Mater. Interfaces 2017, 9, 43021. 29140676 10.1021/acsami.7b12151

[14] Y. Yu, S. Y. Huang, Y. Li, S. N. Steinmann, W. Yang, L. Cao, Nano Lett. 2014, 14, 553. 24397410 10.1021/nl403620g

[15] G. Li, Z. Chen, Y. Li, D. Zhang, W. Yang, Y. Liu, L. Cao, ACS Nano 2020, 14, 1707. 31944096 10.1021/acsnano.9b07324

[16] C. C. Cheng, A. Y. Lu, C. C. Tseng, X. Yang, M. N. Hedhili, M. C. Chen, K. H. Wei, L. J. Li, Nano Energy 2016, 30, 846.

[17] Y. He, P. Tang, Z. Hu, Q. He, C. Zhu, L. Wang, Q. Zeng, P. Golani, G. Gao, W. Fu, Z. Huang, C. Gao, J. Xia, X. Wang, X. Wang, C. Zhu, Q. M. Ramasse, A. Zhang, B. An, Y. Zhang, S. Marti‐Sanchez, J. R. Morante, L. Wang, B. K. Tay, B. I. Yakobson, A. Trampert, H. Zhang, M. Wu, Q. J. Wang, J. Arbiol, Z. Liu, Nat. Commun. 2020, 11, 57. 31896753 10.1038/s41467-019-13631-2 PMC6940382

[18] S. Boandoh, F. O. T. Agyapong‐Fordjour, S. H. Choi, J. S. Lee, J. H. Park, H. Y. Ko, G. Han, S. J. Yun, S. Park, Y. M. Kim, W. Yang, Y. H. Lee, K. K. Kim, ACS Appl. Mater. Interfaces 2019, 11, 1579. 30525400 10.1021/acsami.8b16261

[19] T. Sun, H. Zhang, X. Wang, J. Liu, C. Xiao, S. U. Nanayakkara, J. L. Blackburn, M. V. Mirkin, E. M. Miller, Nanoscale Horiz. 2019, 4, 619.

[20] X. Huang, M. Leng, W. Xiao, M. Li, J. Ding, T. L. Tan, W. S. V. Lee, J. Xue, Adv. Funct. Mater. 2016, 27, 1604943.

[21] J. Guo, C. Liu, Y. Sun, J. Sun, W. Zhang, T. Si, H. Lei, Q. Liu, X. Zhang, J. Solid State Chem. 2018, 263, 84.

[22] S. Bolar, S. Shit, J. S. Kumar, N. C. Murmu, R. S. Ganesh, H. Inokawa, T. Kuila, Appl. Catal., B 2019, 254, 432.

[23] J. Yan, A. Rath, H. Wang, S. H. Yu, S. J. Pennycook, D. H. C. Chua, Mater. Res. Lett. 2019, 7, 275.

[24] Z. Lin, B. R. Carvalho, E. Kahn, R. Lv, R. Rao, H. Terrones, M. A. Pimenta, M. Terrones, 2D Mater. 2016, 3, 022002.

[25] J. Wang, F. Xu, H. Jin, Y. Chen, Y. Wang, Adv. Mater. 2017, 29, 1605838. 10.1002/adma.201605838 28234409

[26] T. F. Jaramillo, K. P. Jorgensen, J. Bonde, J. H. Nielsen, S. Horch, I. Chorkendorff, Science 2007, 317, 100. 17615351 10.1126/science.1141483

[27] B. Song, S. K. Yun, J. Jiang, K. Beach, W. Choi, Y. Kim, H. Terrones, Y. J. Song, D. L. Duong, Y. H. Lee, arXiv:2002.07333 2002.

[28] F. O. T. Agyapong‐Fordjour, S. Oh, J. Lee, S. Chae, K. H. Choi, S. H. Choi, S. Boandoh, W. Yang, J. Huh, K. K. Kim, J. Y. Choi, ACS Appl. Energy Mater. 2019, 2, 5785. 10.1021/acsami.8b16261 30525400

[29] X. Huang, M. Leng, W. Xiao, M. Li, J. Ding, T. L. Tan, W. S. V. Lee, J. Xue, Adv. Funct. Mater. 2017, 27, 160494.

[30] J. Shi, X. Wang, S. Zhang, L. Xiao, Y. Huan, Y. Gong, Z. Zhang, Y. Li, X. Zhou, M. Hong, Q. Fang, Q. Zhang, X. Liu, L. Gu, Z. Liu, Y. Zhang, Nat. Commun. 2017, 8, 958. 29038430 10.1038/s41467-017-01089-z PMC5643402

[31] D. Voiry, H. Yamaguchi, J. Li, D. C. Alves, T. Fujita, M. Chen, T. Asefa, V. B. Shenov, G. Eda, M. Chhowalla, Nat. Mater. 2013, 12, 850. 23832127 10.1038/nmat3700

[32] M. Yan, X. Pan, P. Wang, F. Chen, L. He, G. Jiang, J. Z. Liu, X. Xu, X. Liao, J. Yang, L. mai, Nano Lett. 2017, 17, 4109. 28585826 10.1021/acs.nanolett.7b00855

[33] J. Zhang, W. Xiao, P. Xi, S. Xi, Y. Du, D. Gao, J. Ding, ACS Energy Lett. 2017, 2, 1022.

[34] Y. Huang, Y. Sun, X. Zheng, T. Aoki, B. Pattengale, J. Huang, X. He, W. Bian, S. Younan, N. Williams, J. Hu, J. Ge, N. Pu, X. Yan, X. Pan, L. Zhang, Y. Wei, Nat. Commun. 2019, 10, 982. 30816110 10.1038/s41467-019-08877-9 PMC6395606

[35] J. Yang, Y. Wang, M. J. Lagos, V. Manichev, R. Fullon, X. Song, D. Voiry, S. Chakraborty, W. Zhang, P. E. Batson, L. Feldman, T. Gustafsson, M. Chhowalla, ACS Nano 2019, 13, 9958. 31398001 10.1021/acsnano.9b05226

[36] C. T. Koch, Ph.D. Thesis, Arizona State University 2002.

[37] A. R. Jadhav, K. Ashwani, J. Lee, T. Yang, S. Na, J. Lee, Y. Luo, X. Liu, Y. Hwang, Y. Liu, H. Lee, J. Mater. Chem. A 2020, 8, 24501.

[38] G. Kress, J. Furthmüller, Phys. Rev. B 1996, 54, 11169. 10.1103/physrevb.54.11169 9984901

[39] J. P. Perdew, K. Burke, M. Ernzerhof, Phys. Rev. Lett. 1996, 77, 3865. 10062328 10.1103/PhysRevLett.77.3865

[40] G. Román‐Pérez, J. M. Soler, Phys. Rev. Lett. 2009, 103, 096102. 19792809 10.1103/PhysRevLett.103.096102

[41] P. E. Blöchl, Phys. Rev. B 1994, 50, 17953. 10.1103/physrevb.50.17953 9976227

[42] J. K. Nørskov, T. Bligaard, A. Logadottir, J. R. Kitchin, J. G. Chen, S. Pandelov, U. Stimming, J. Electrochem. Soc. 2005, 152, J23.

