# Supplementary material for: Substitutional Vanadium Sulfide Nanodispersed in MoS2 Film for Pt‐Scalable Catalyst
Source: Adv Sci (Weinh). 2021 Jun 3;8(16):2003709. doi: 10.1002/advs.202003709 (PMC8373103; doi:10.1002/advs.202003709)
Supplement: Supplementary file 1 — Supporting Information [file ADVS-8-2003709-s001.pdf]

WILEY-VCH

© 2021 Wiley-VCH GmbH

ADVANCED SCIENCE
Open Access

Supporting Information

for *Adv. Sci.*, DOI: 10.1002/advs.202003709

Substitutional Vanadium Sulfide Nanodispersed in MoS_2 Film for Pt-Scalable Catalyst

Frederick Osei-Tutu Agyapong-Fordjour, Seok Joon Yun, Hyung-Jin Kim, Wooseon Choi, Balakrishnan Kirubasankar, Soo Ho Choi, Laud Anim Adofo, Stephen Boandoh, Yong In Kim, Soo Min Kim, Young-Min Kim, Young Hee Lee, Young-Kyu Han*, and Ki Kang Kim**

Copyright WILEY-VCH Verlag GmbH & Co. KGaA, 69469 Weinheim, Germany, 2018.

Supporting Information

Substitutional Vanadium Sulfide Nanodispersed in MoS_2 Film for Pt-Scalable Catalyst

Frederick Osei-Tutu Agyapong-Fordjour, Seok Joon Yun, Hyung-Jin Kim, Wooseon Choi, Balakrishnan Kirubasankar, Soo Ho Choi, Laud Anim Adofo, Stephen Boandoh, Yong In Kim, Soo Min Kim, Young-Min Kim, Young Hee Lee, Young-Kyu Han*, and Ki Kang Kim**

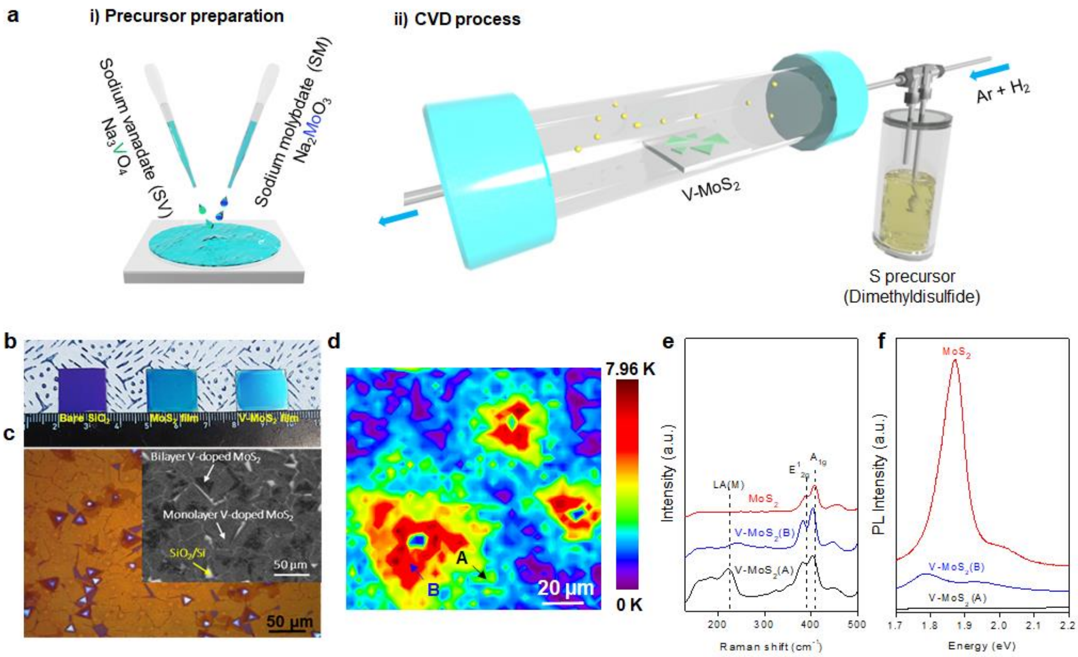

Figure S1. CVD growth procedure and optical characterization of V-MoS$_2$. a) Schematic of i) precursor preparation and ii) chemical vapor deposition for growing V-MoS$_2$ on SiO$_2$/Si substrate. b) Photograph of bare SiO$_2$/Si and an as-grown MoS$_2$ and V-MoS$_2$ film on SiO$_2$/Si substrate. c) Optical microscopy image of monolayer V-MoS$_2$ on SiO$_2$/Si substrate. Inset: SEM image of V-MoS$_2$ film showing V-MoS$_2$ film is predominantly monolayer with a small multilayer portion. d) Raman mapping image of V-MoS$_2$ for A_{1g} mode (~398.4 cm^{-1}). Different colors in the mapping image indicate different thicknesses of V-MoS$_2$. e,f) Raman and PL spectra for pure MoS$_2$ and V-MoS$_2$ at positions A and B in d). The characteristic Raman peak of V-MoS$_2$ is newly developed at 225 cm^{-1} and quenching of PL intensity as a result of the formation of metallic VS$_n$ units in MoS$_2$. [1]

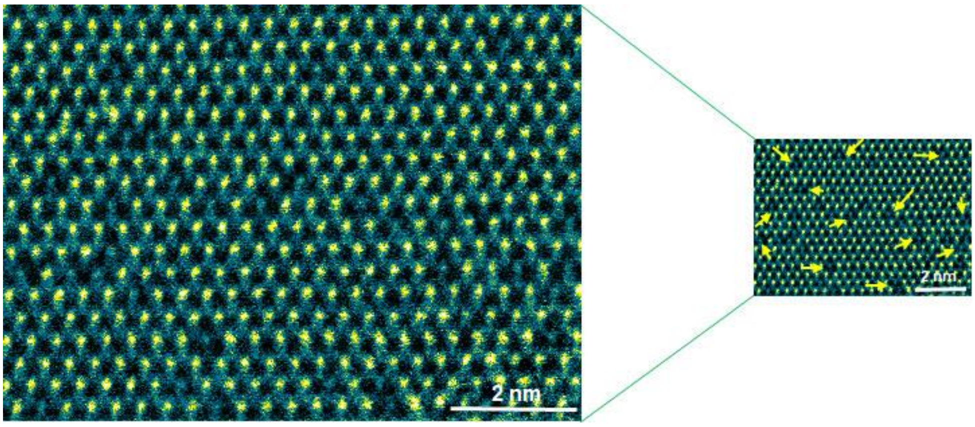

Figure S2. STEM image for VS_n dispersion in V-MoS_2. The zoomed-out STEM image of V-MoS_2 clearly shows the nanodispersed VS_n units indicated by yellow arrows.

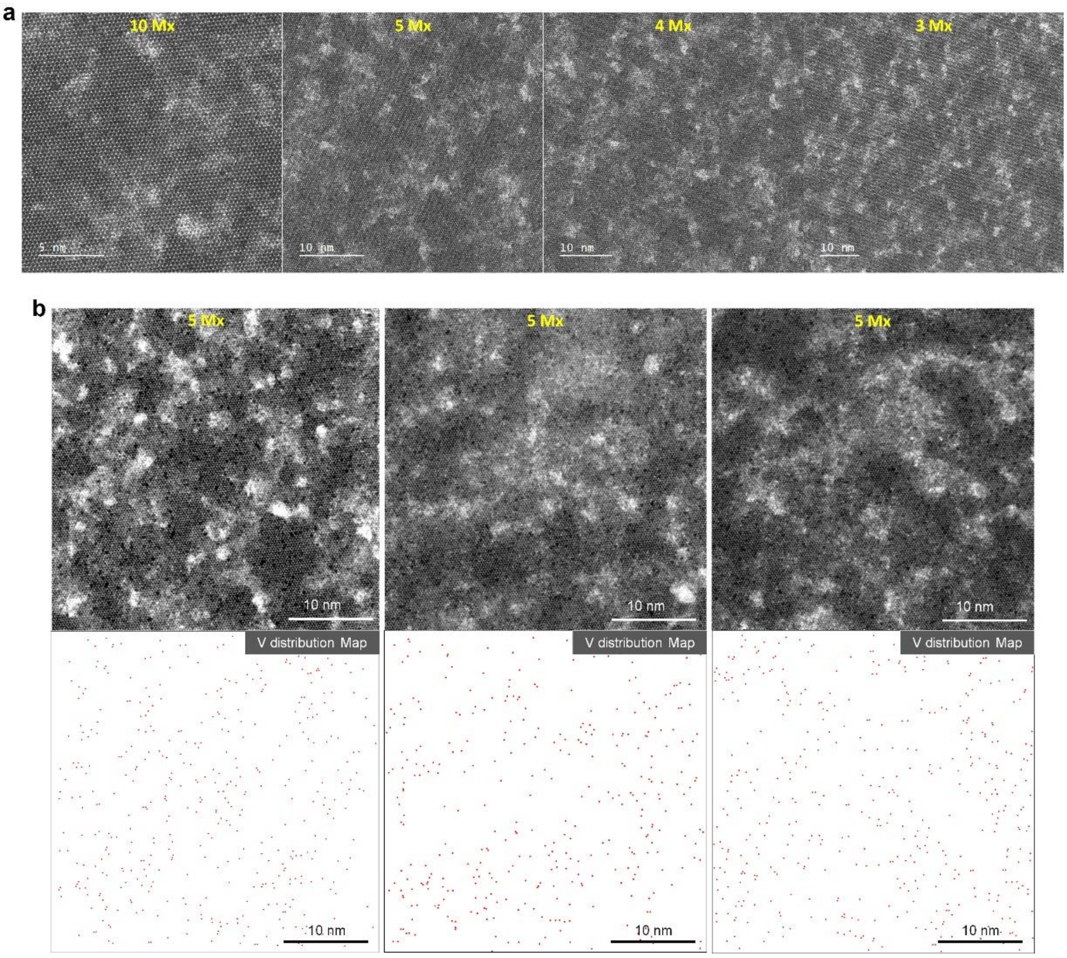

Figure S3. Uniformity of VS_n units in V-MoS_2 lattice. a) STEM images of $V_{(9.3\%)}$-MoS_2 with various large field of views. The V-substitutions displayed as dark spots at Mo sites in V-MoS_2 are clearly observed in 10 Mx and 5 Mx images. Beyond 5 Mx could not identify V atoms. b) 5 Mx STEM images and corresponding V-distribution maps from three different regions. V atoms are homogenously distributed in MoS_2 lattice with scarce V aggregation formation. The white clusters on MoS_2 surface stemmed probably from PMMA residue which was used during the transfer process. [2]

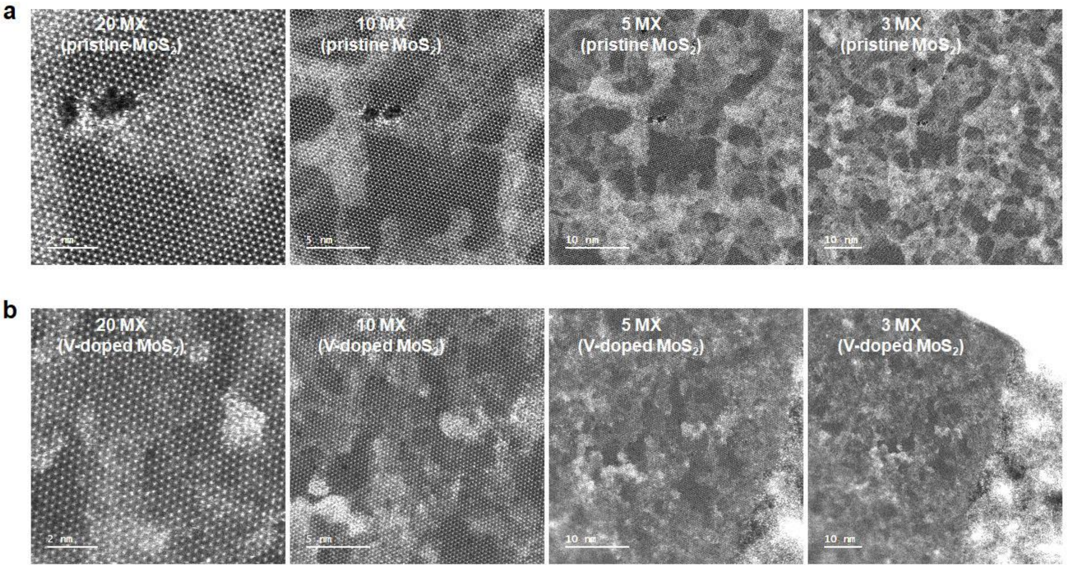

Figure S4. **STEM images of** a) pristine and b) V-doped MoS_2 with various magnification views. Bright contrast features are seen in both samples, indicating that those features are not from the presence of V-clusters.

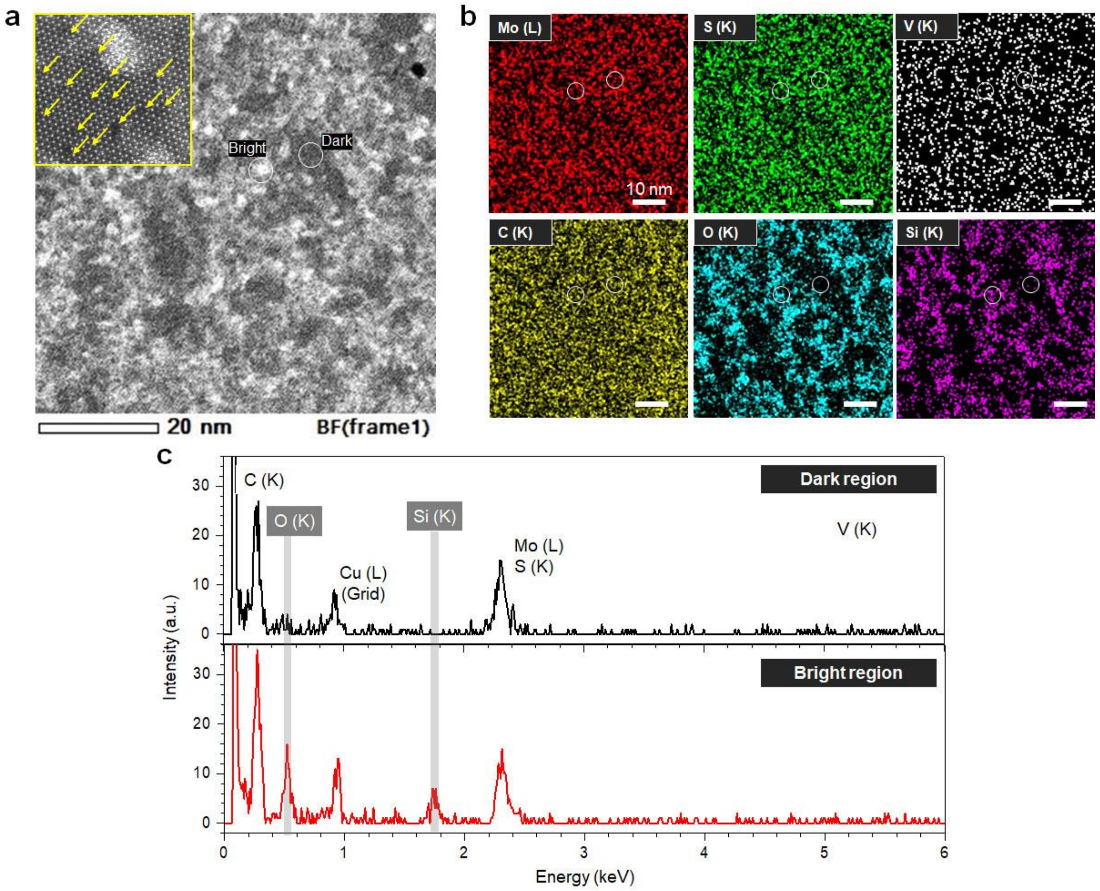

Figure S5. Elemental analysis of V-doped MoS_2. a) STEM and b) corresponding EDS mapping images of transferred V-doped MoS_2. c) EDS spectra extracted from bright and dark regions in a. The yellow arrows in the inset indicate V atoms in MoS_2 lattice. The shape of bright contrast features in ADF-STEM image does not match with Mo, S, V, and C elements in the corresponding EDS mapping images, further confirming the bright features are not from V clusters. O and Si elements are closely correlated from EDX mapping and spectra as well corresponding to the distribution of the bright contrast features.

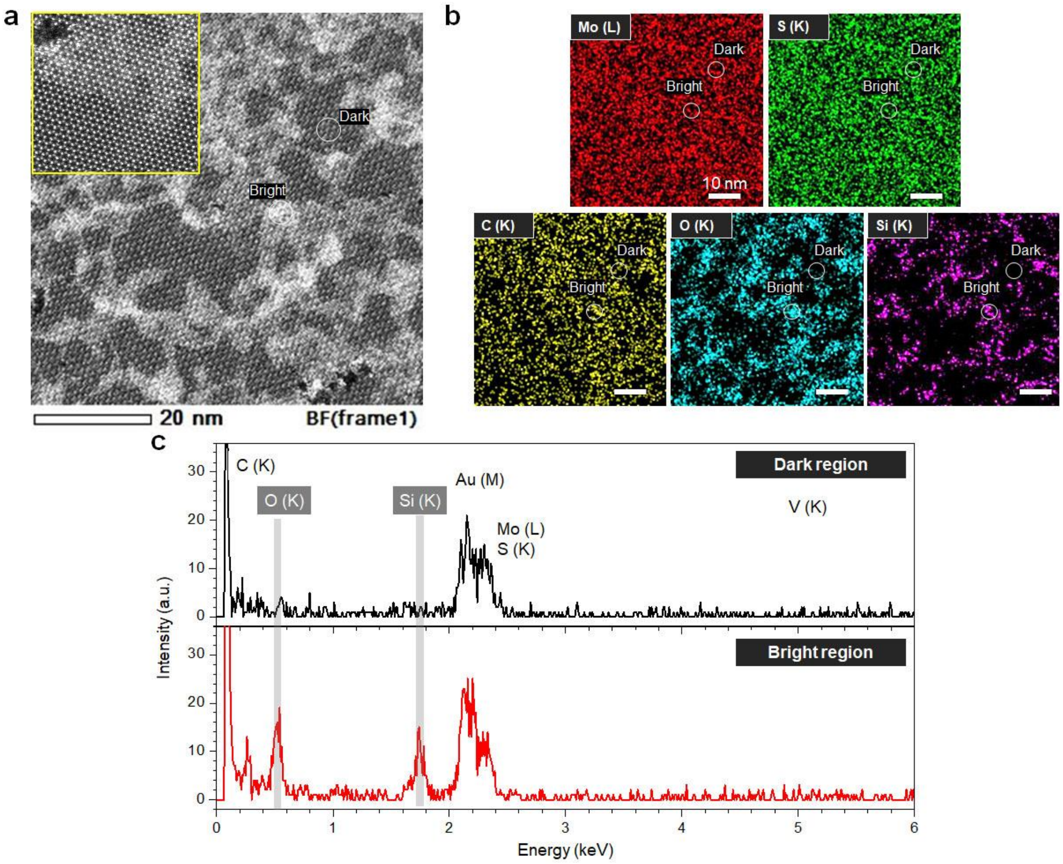

Figure S6. Elemental analysis of pristine MoS_2. a) STEM and b) EDS mapping images of transferred pristine MoS_2. c) EDS spectra extracted from bright and dark regions in a. O and Si elements are closely correlated from EDX mapping and spectra as well corresponding to the distribution of the bright contrast features. Therefore, we conclude that the bright contrast feature is probably related to PMMA residue introduced for transfer of the samples.

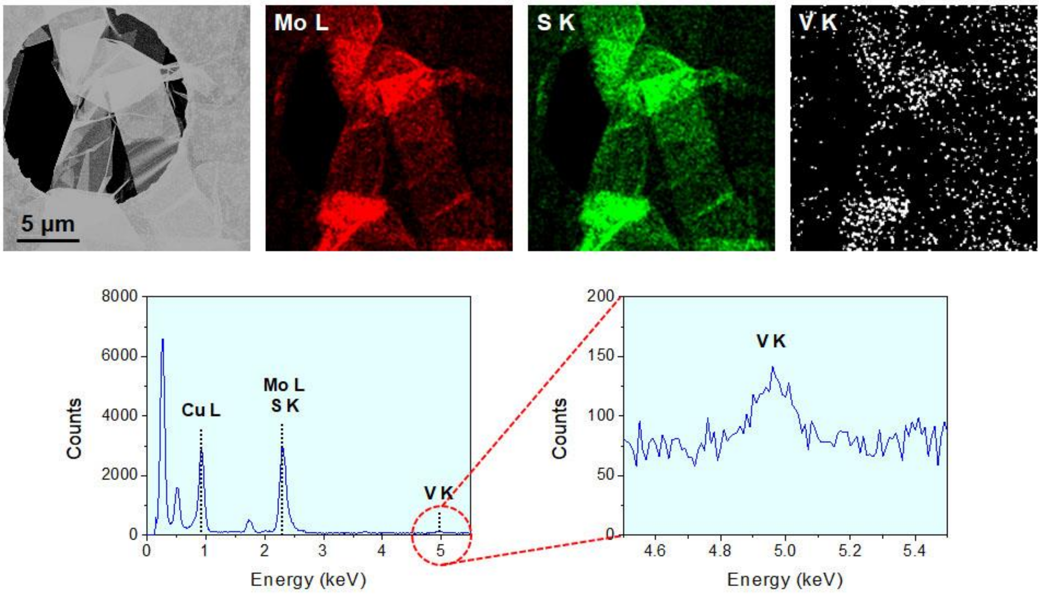

Figure S7. Energy dispersive X-ray spectroscopy analysis of V-MoS_2. The EDS mapping images and spectrum for V-MoS_2 prove the presences of Mo, S, and V atoms.

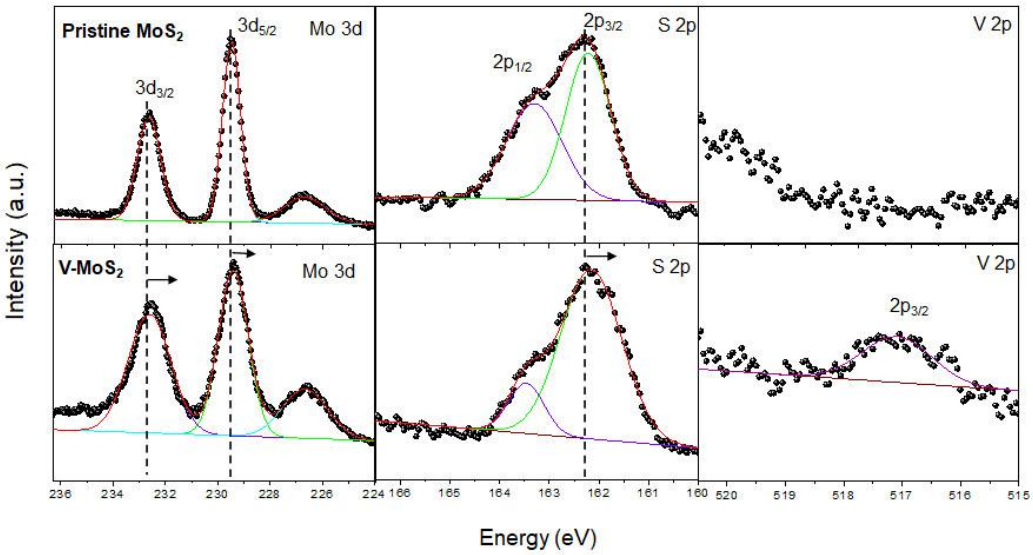

Figure S8. X-ray photoelectron spectroscopy (XPS) analysis of MoS_2 and V-MoS_2. XPS core-level spectra of Mo 3d, S 2p, and V 2p of MoS_2 and V-MoS_2, respectively. The two Mo 3d peaks and two S 2p peaks are Mo^{4+} $3d_{5/2}$, Mo^{4+} $3d_{3/2}$, S $2p_{3/2}$, and S $2p_{1/2}$ originating from MoS_2. The V^{4+} $2p_{3/2}$ peak is clearly observed in V-MoS_2, implying the presence of V atoms in V-MoS_2. The slight red shift of the Mo 3d and S 2p peaks are attributed to the *p*-doping effect of V. [3]

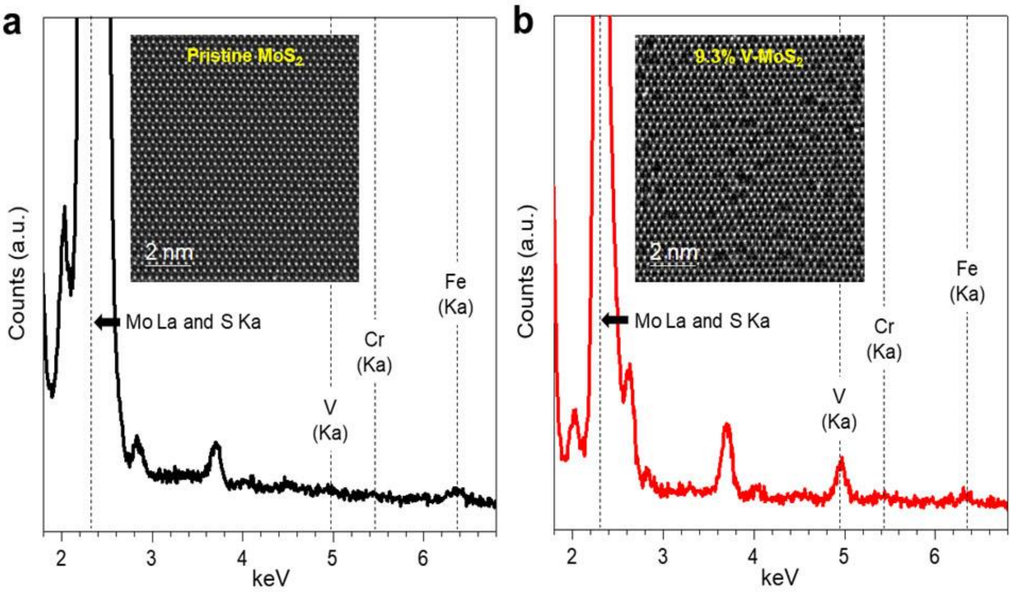

Figure S9. Presence of V atoms in MoS_2 lattice. EDX spectra for a) pristine and b) V-MoS_2 with representative STEM images. While dark spots (V atoms) in STEM image of pristine MoS_2 are rarely found, the distinct spots are observed in V-MoS_2. Furthermore, the prominent V Ka peak is clearly visible in the EDX spectrum of V-MoS_2. The tiny Cr and Fe Ka peaks detected in both EDX spectra probably come from metal parts of objective lens and EDX holder (known as instrumental peaks),[4] rather than impurities in the MoS_2 lattice.[5,6] Therefore, the dark spots are V atoms, not Cr or Fe atoms.

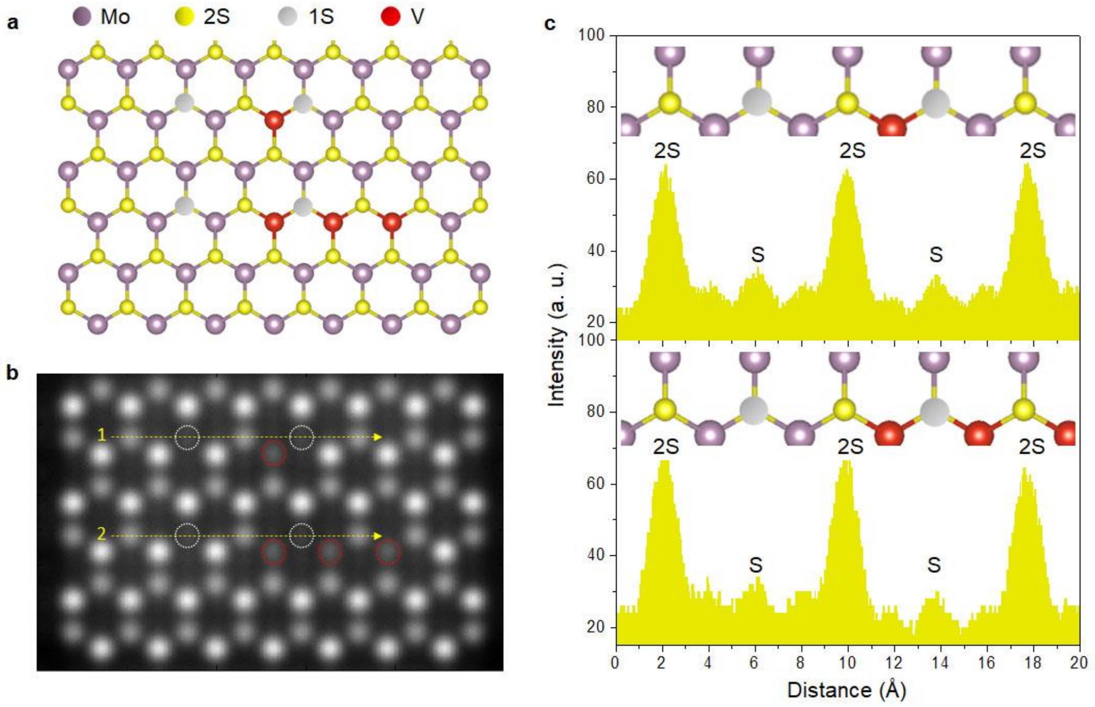

Figure S10. Intensity profile of 1S and 2S next to Mo and V atoms. a) Schematic ball-and-stick model of V-MoS_2 with Vac_s and b) corresponding simulated image. c) Intensity profiles of 1S and 2S atoms along lines 1 and 2 in b. Even though 1S and 2S next to V atom are slightly dimmer compared to those next Mo, they are still clearly distinguished by the intensity.

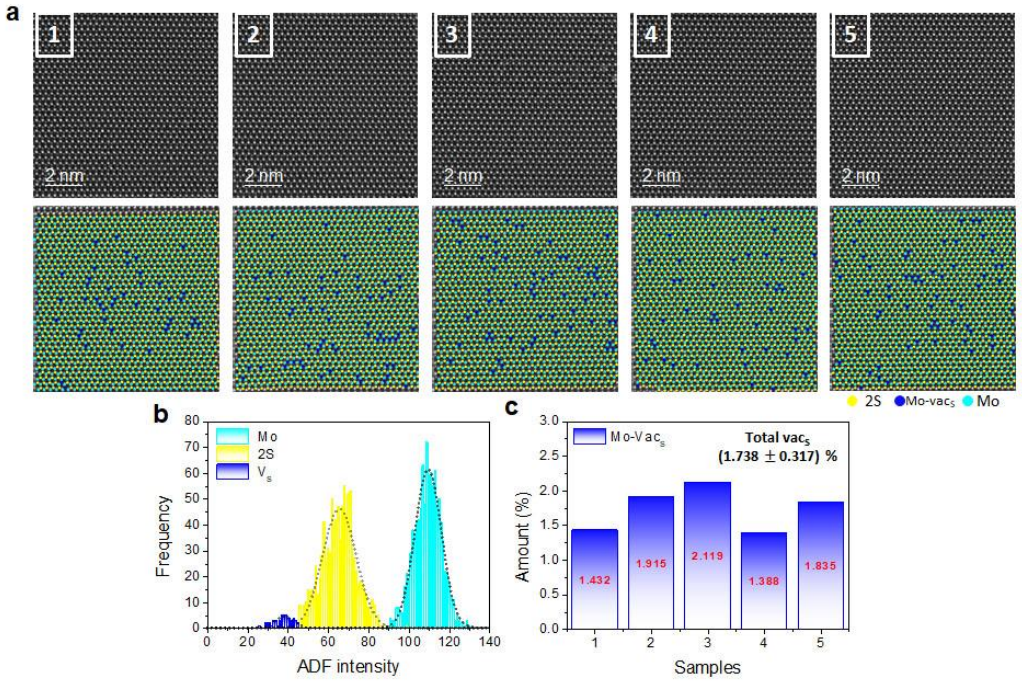

Figure S11. Atom site-wise mapping of pristine MoS_2 monolayer. a) Weiner-filtered ADF-STEM images (top) and false-colored STEM images (bottom) of MoS_2. b) Frequency of Mo, S, and vac_s sites with their ADF intensities. c) Atomic % of sulfur vacancies in pristine MoS_2 (Mo-vac_s).

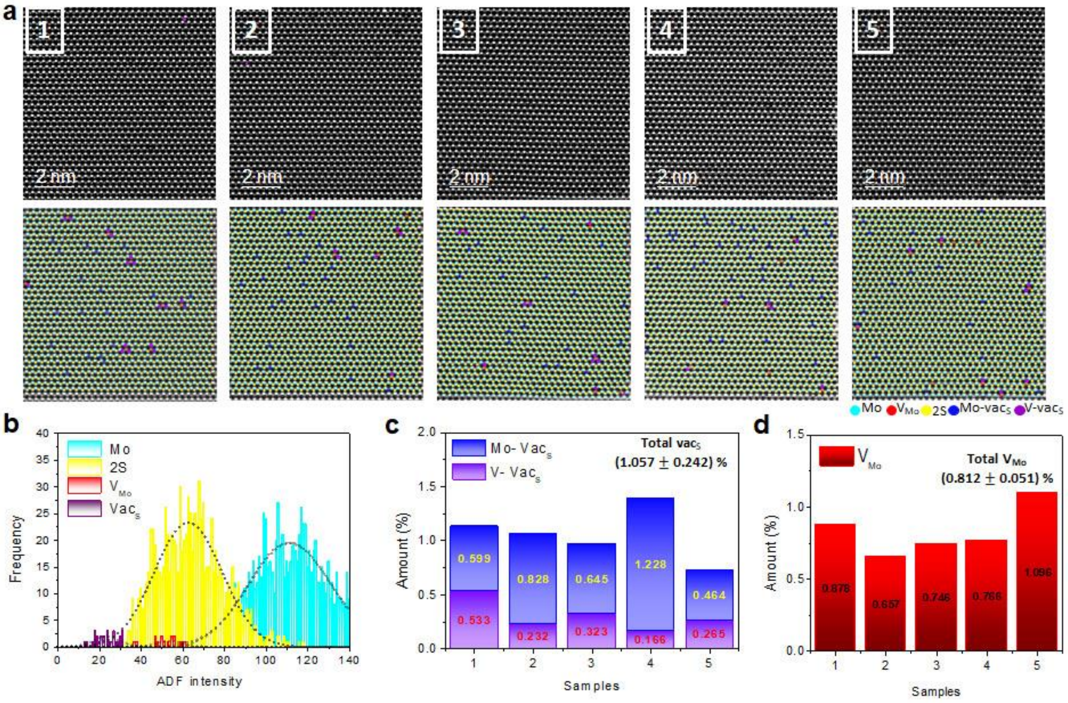

Figure S12. Atom site-wise mapping of 2:1 V-MoS$_2$ (V:Mo). a) Weiner-filtered ADF-STEM images (top) and false-colored STEM images (bottom) of 2:1 V-MoS$_2$. b) Frequency of Mo, S, V_{Mo}, and vac$_s$ sites with their ADF intensities. c,d) Atomic % of Mo-vac$_s$ and V-vac$_s$ and V_{Mo}, respectively.

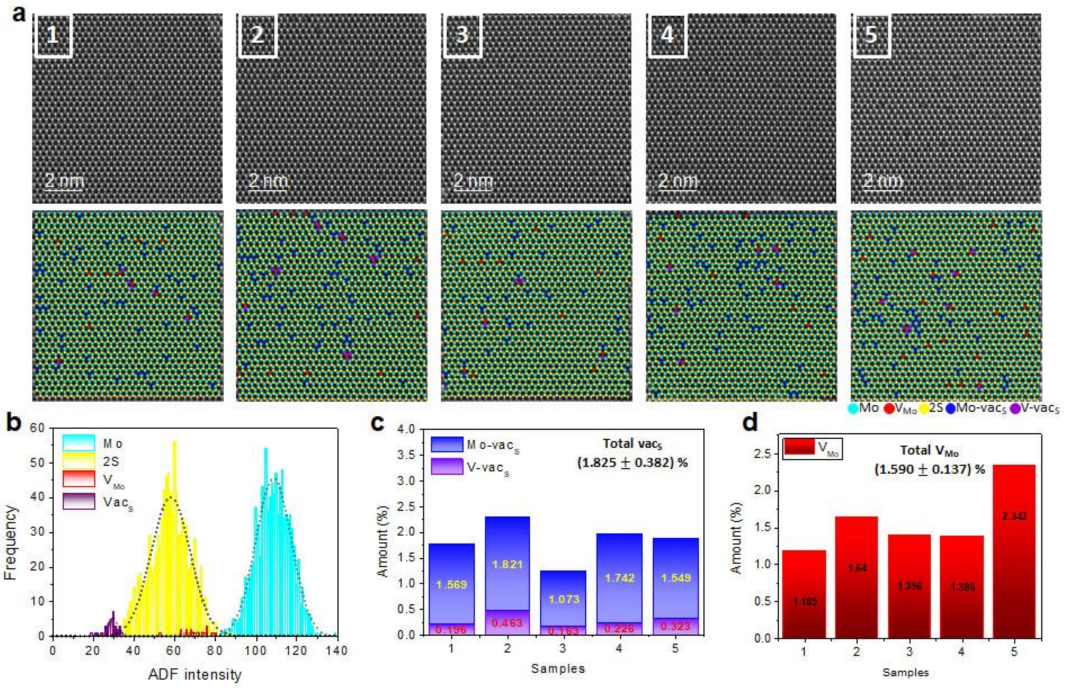

Figure S13. Atom site-wise mapping of 4:1 V-MoS_2 (V:Mo). a) Weiner-filtered ADF-STEM images (top) and false-colored STEM images (bottom) of 4:1 V-MoS_2. b) Frequency of Mo, S, V_{Mo}, and vac_s sites with their ADF intensities. c,d) Atomic % of Mo-vac_s and V-vac_s and V_{Mo}, respectively.

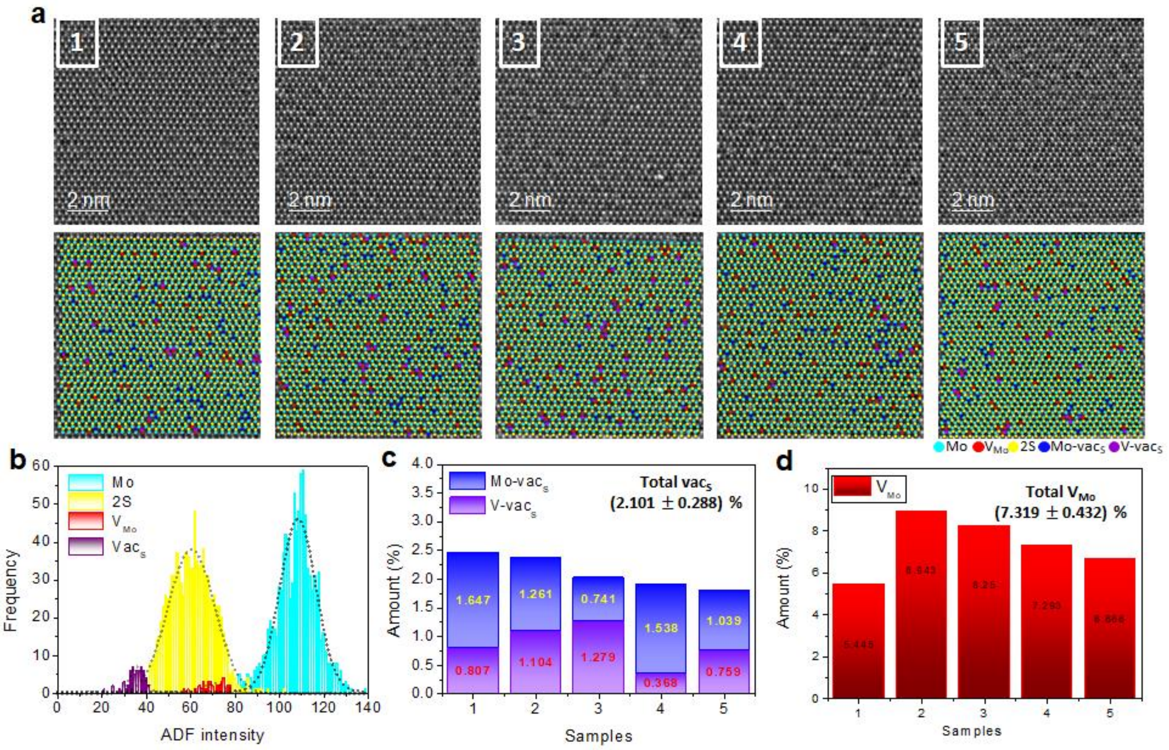

Figure S14. Atom site-wise mapping of 8:1 V-MoS$_2$ (V:Mo). a) Weiner-filtered ADF-STEM images (top) and false-colored STEM images (bottom) of 8:1 V-MoS$_2$. b) Frequency of Mo, S, V_{Mo} and vac$_s$ sites with their ADF intensities. c,d) Atomic % of Mo-vac$_s$ and V-vac$_s$ and V_{Mo}, respectively.

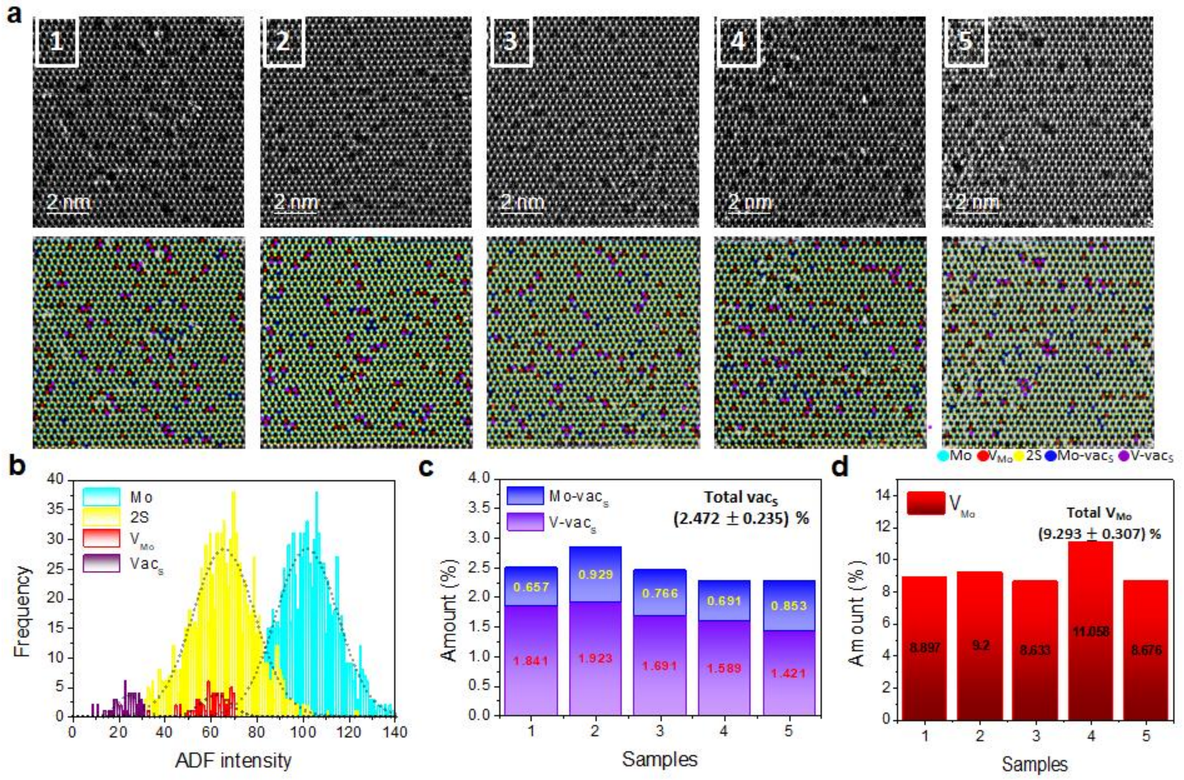

Figure S15. Atom site-wise mapping of 20:1 V-MoS$_2$ (V:Mo). a) Weiner-filtered ADF-STEM images (top) and false-colored STEM images (bottom) of 20:1 V-MoS$_2$. b) Frequency of Mo, S, V$_{Mo}$, and vac$_s$ sites with their ADF intensities. c,d) Atomic % of Mo-vac$_s$ and V-vac$_s$ and V$_{Mo}$, respectively.

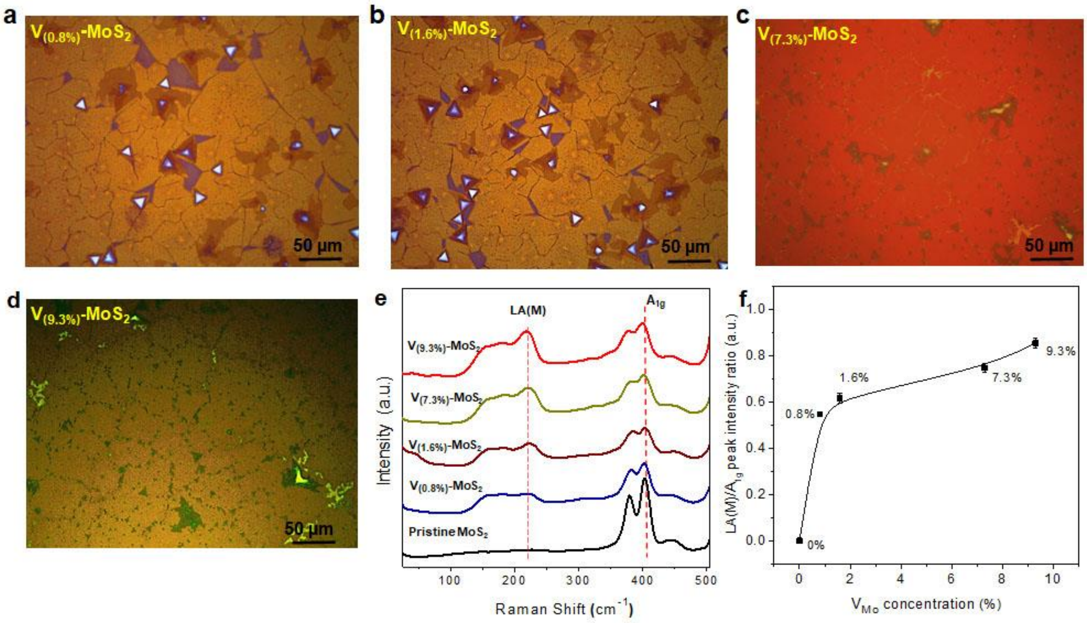

Figure S16. Optical microscopy image and Raman spectra of V-MoS_2 synthesized with different V at%. a-d) Optical microscopy images of as-grown V-MoS_2 film with 0.8, 1.6, 7.3, and 9.3 V at%, respectively. e) Raman spectra of as-grown V-MoS_2 film with various V:Mo mixing ratio. f) Peak intensity ratio of LA(M) mode (~225 cm^{-1}) to A_{1g} as a function of the V concentration.[1]

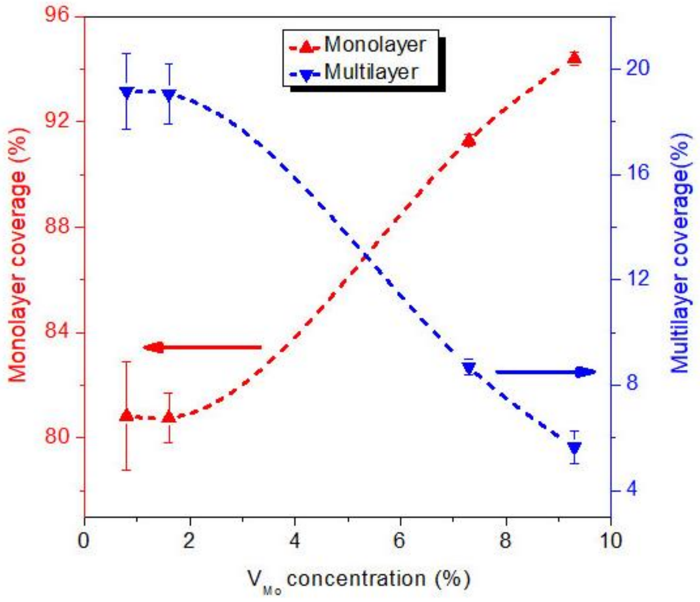

Figure S17. Statistics of monolayer and multilayer extracted from optical images using commercial Gwyddion software.[7]

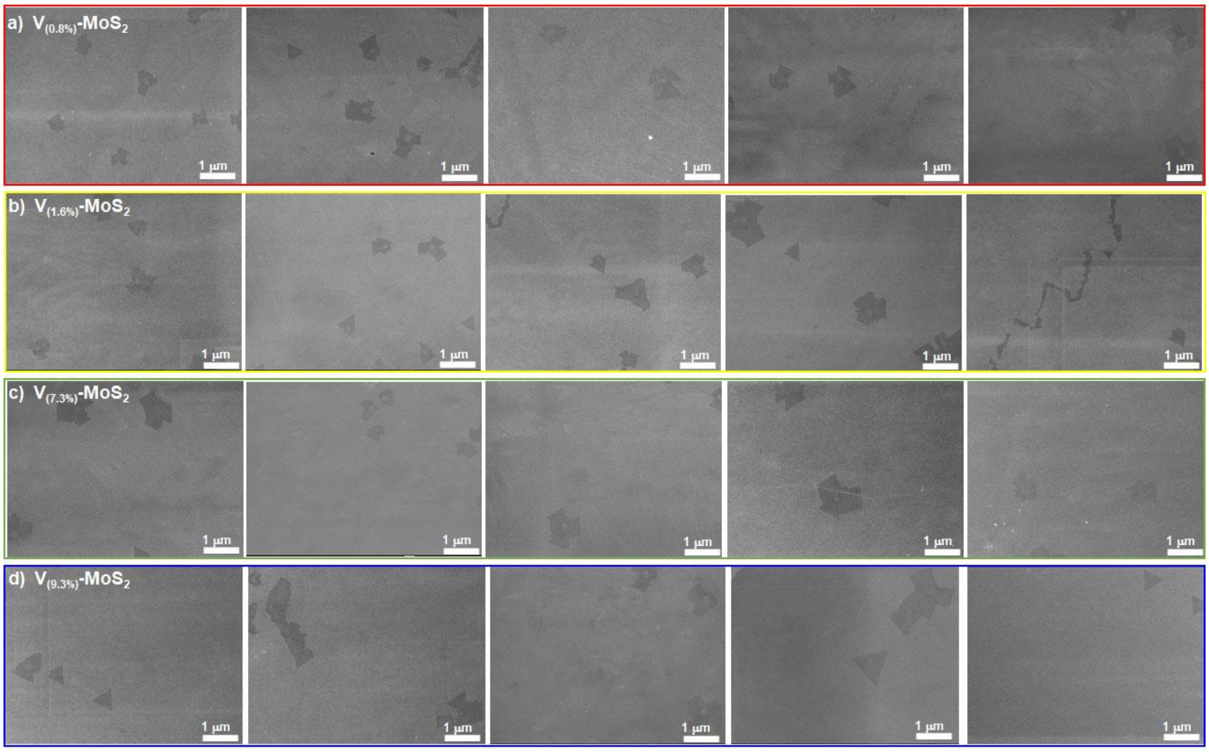

Figure S18. Observation of multilayer (dark) regions in V-MoS$_2$. a-d) SEM images of as-grown V-MoS$_2$ film with different V concentrations of 0.8, 1.6, 7.3, and 9.3 V at%.

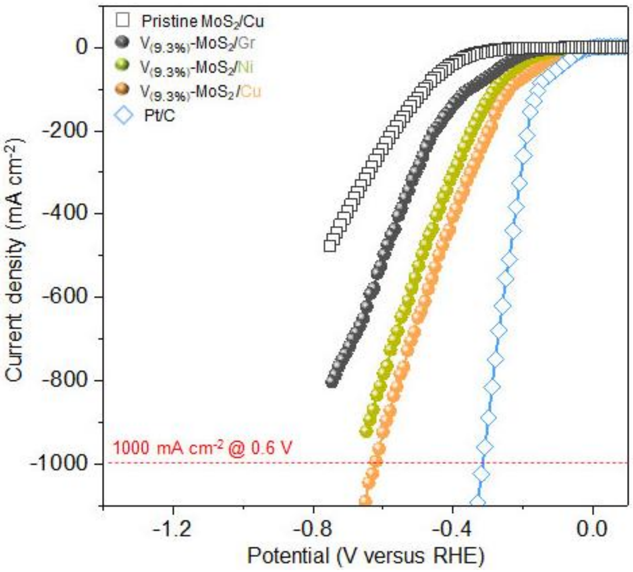

Figure S19. High current density of V-MoS_2 catalyst on various substrate. The high current density exceeding 1,000 mA cm^{-2} at 0.6 V for $V_{(9.3\%)}$-MoS_2/Cu demonstrates its suitability for industrial application.

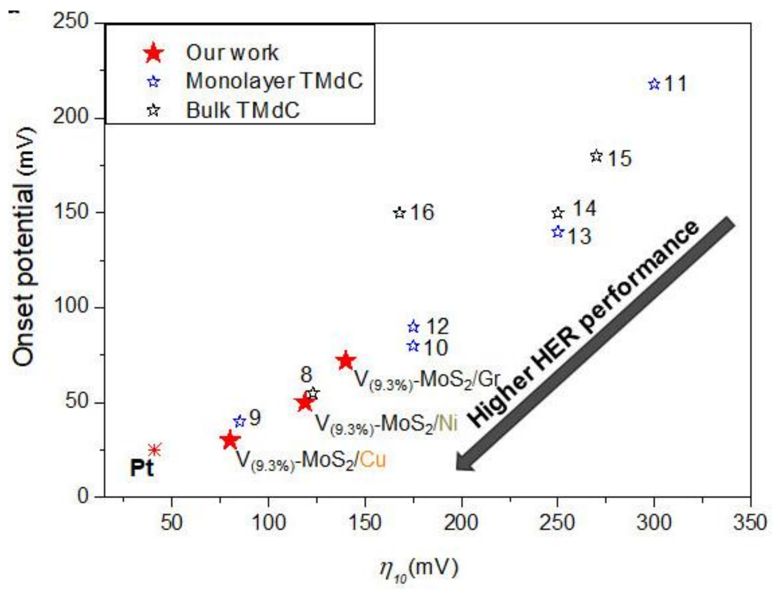

Figure S20. Comparison of electrocatalytic parameters of V-MoS_2 film with those of other 2D TMdCs. Onset potential versus overpotential for 10 mA cm^{-2} current density (η_{10}) of our materials compared with other 2D electrocatalysts. $V_{(9.3\%)}$-MoS_2 film on the Cu substrate exhibits extremely low onset potential of -30 mV, similar to that of Pt (-25 mV). Comparative data were collected from the literatures.[8-16]

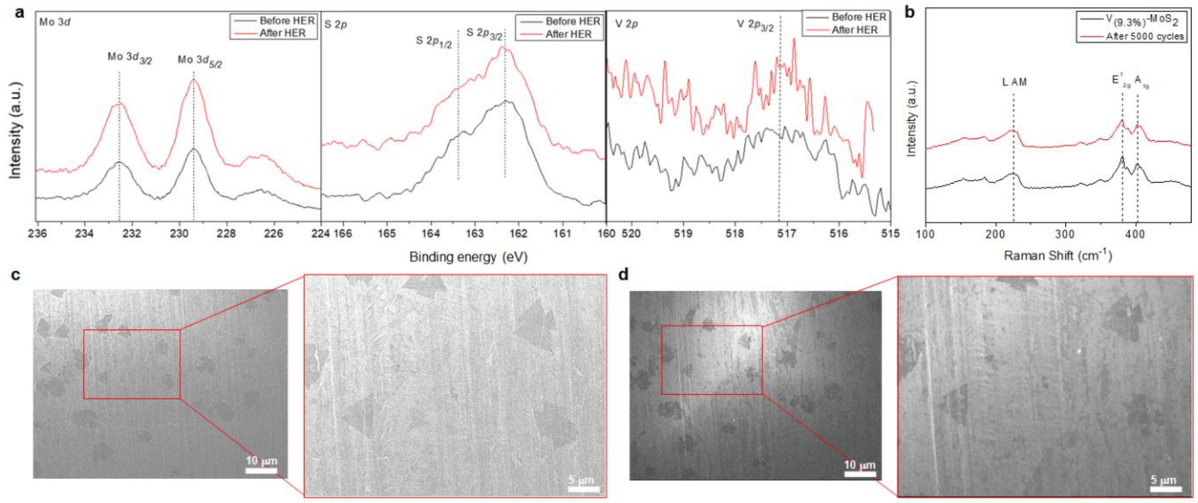

Figure S21. Stability of $V_{(9.3\%)}$-MoS_2 before and after HER cycling test. a) XPS core-level spectra of $V_{(9.3\%)}$-MoS_2 before and after HER cycling-test for Mo 3d, S 2p, and V 2p, respectively. b) Representative Raman spectra analysis of $V_{(9.3\%)}$-MoS_2 before and after 5000 HER cycling test. c,d) SEM image of V-MoS_2 c) before and d) after 5000 cycling with corresponding zoomed-in images. No significant changes are observed in the XPS core-level spectra, Raman spectra, and SEM images after HER cycle test. These indicate that V-MoS_2 is stable during HER.

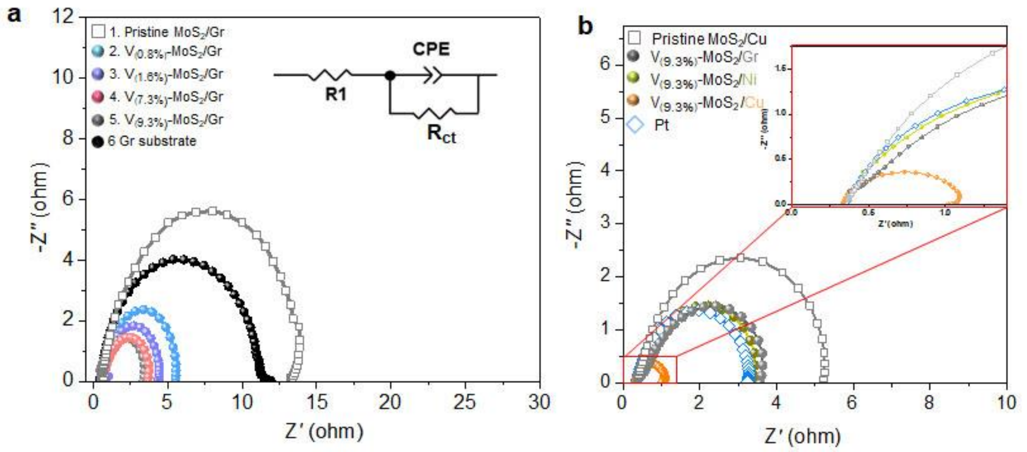

Figure S22. Charge transfer resistance analysis of V-MoS_2 in terms of the V concentration and the substrate. a,b) Nyquist plots for V-MoS_2/Gr and $V_{(9.3\%)}$-MoS_2 on Gr, Ni, and Cu compared to Pt. Inset: The equivalent Randel circuit model is used to extract the series (R1) and charge transfer resistance (R_{ct}).

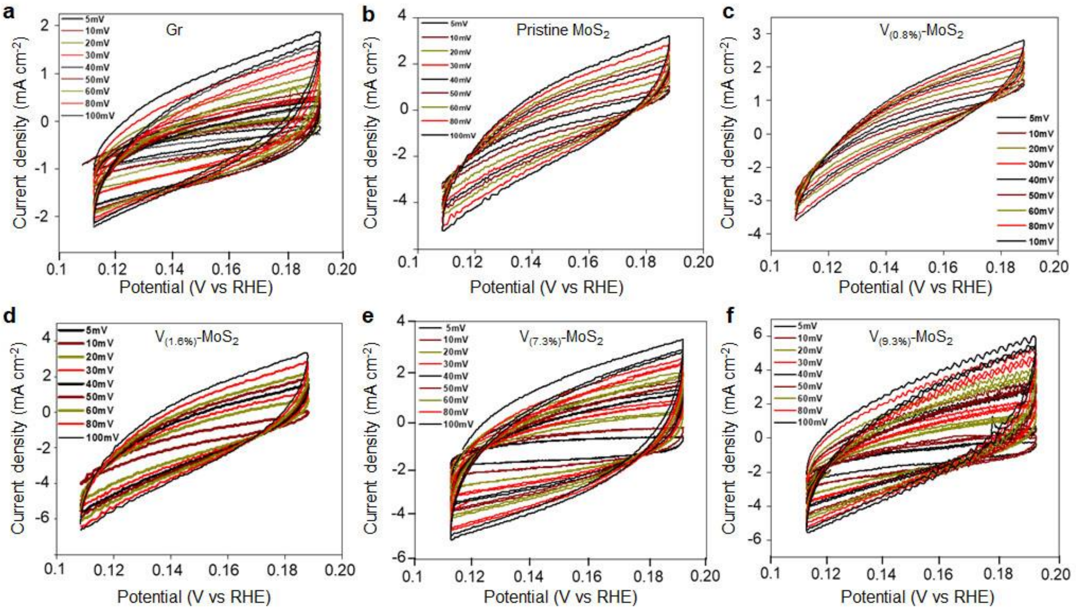

Figure S23. Cyclic voltammetry measurement for double-layer capacitance. a-f) Cyclic voltammograms of Gr, pristine MoS_2, $V_{(0.8\%)}$-MoS_2, $V_{(1.6\%)}$-MoS_2, $V_{(7.3\%)}$-MoS_2, and $V_{(9.3\%)}$-MoS_2 on Gr.

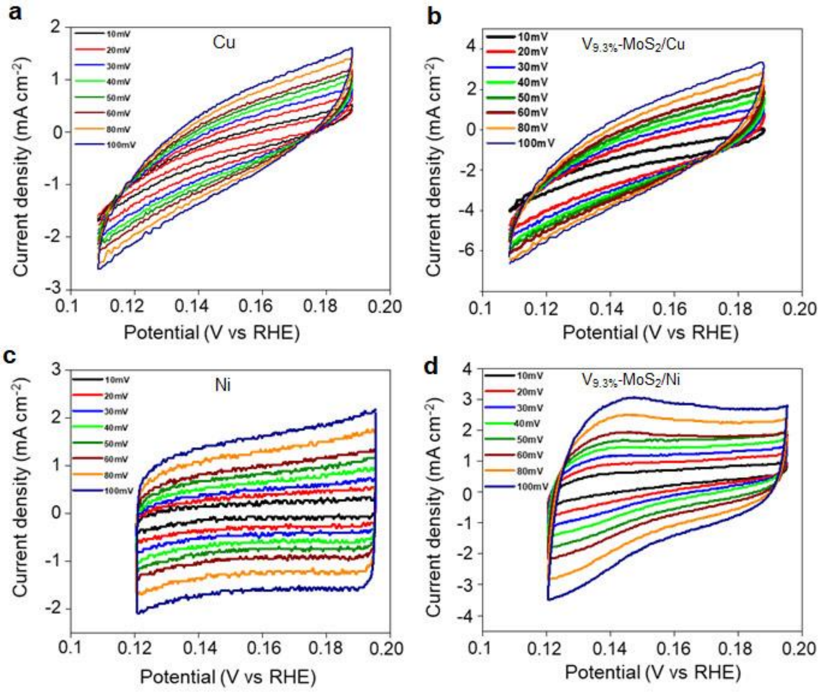

Figure S24. Cyclic voltammetry measurement for double-layer capacitance. a-d) Cyclic voltammograms of bare Cu, $V_{(9.3\%)}$-MoS_2/Cu, bare Ni, and $V_{(9.3\%)}$-MoS_2/Ni, respectively.

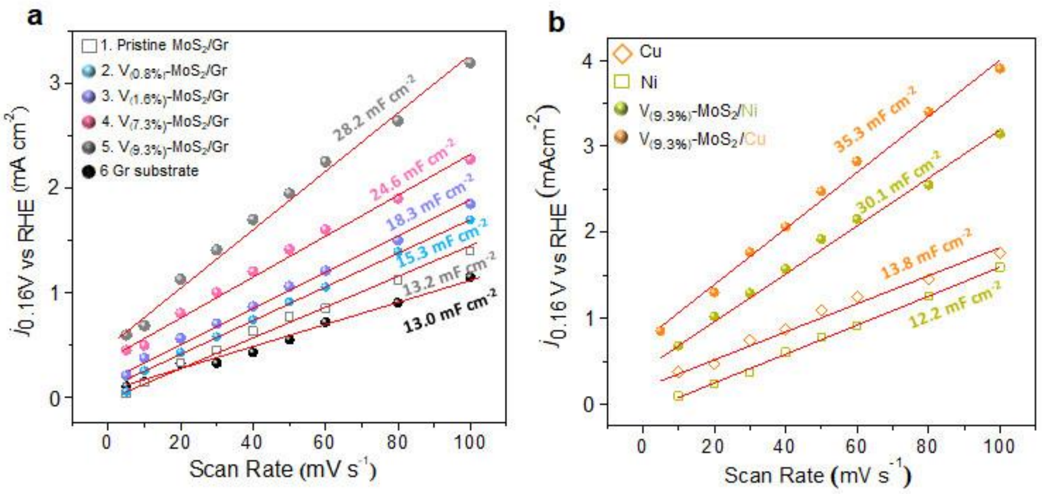

Figure S25. Double-layer capacitance of V-MoS_2 with respect to the V concentration and the substrates. a,b) Capacitive current of V-MoS_2 on Gr substrate for various V concentrations and $V_{(9.3\%)}$-MoS_2 on Cu and Ni substrates as a function of the scan rate. The capacitive current was extracted at 0.15 V vs RHE in Figures S20 and S21, Supporting Information.

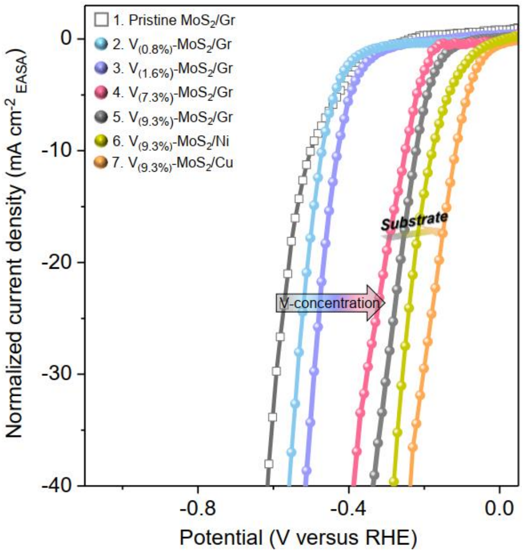

Figure S26. Relative EASA normalized polarization curves for pristine MoS_2 on graphite (Gr) substrate, $V_{(0.8\%)}$-MoS_2/Gr, $V_{(1.6\%)}$-MoS_2/Gr, $V_{(7.3\%)}$-MoS_2/Gr, $V_{(9.3\%)}$-MoS_2/Gr, $V_{(9.3\%)}$-MoS_2/Ni, $V_{(9.3\%)}$-MoS_2/Cu.

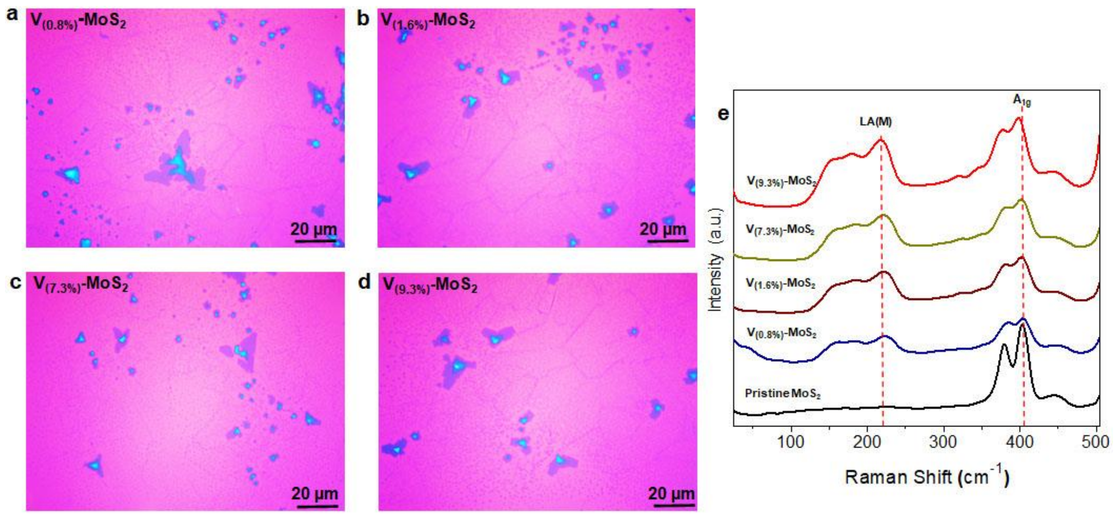

Figure S27. **Optical microscopy images and Raman spectra of newly synthesized V-MoS_2 with different V at%**. a-d) Optical microscopy images of transferred V-MoS_2 film with 0.8, 1.6, 7.3, and 9.3 V at%, respectively. e) Raman spectra of transferred V-MoS_2 film with various V:Mo mixing ratios. The intensity of LA(M) mode is proportional to V-doping concentration which is same trend with previous samples, indicating reproducible V-doping.

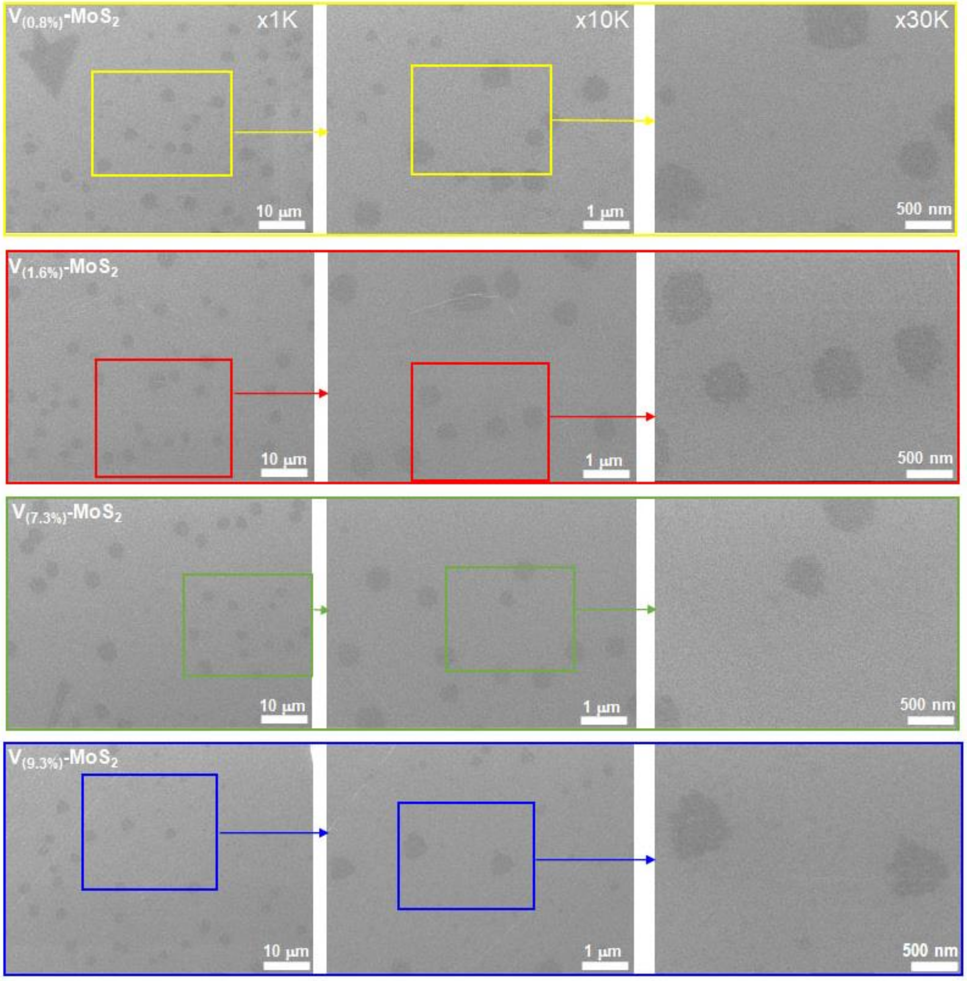

Figure S28. **Observation of multilayer (dark) regions in newly synthesized V-MoS$_2$.** a-d) SEM images of transferred V-MoS$_2$ film at different manifications.

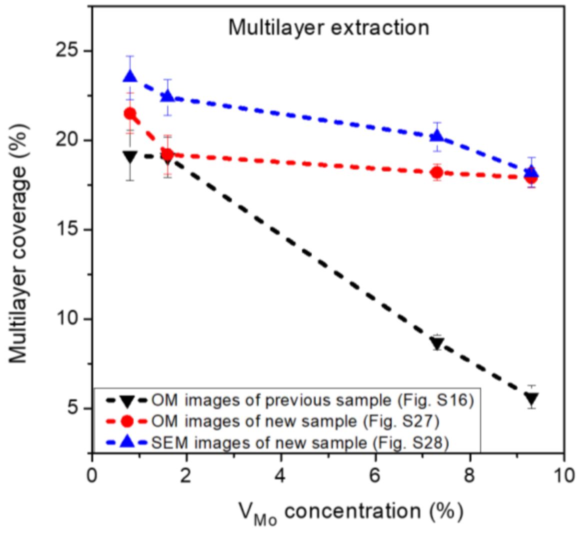

Figure S29. Statistics of multilayer in previous and new V-MoS_2 samples. The data is extracted from optical microscopy and scanning electron microscopy images.

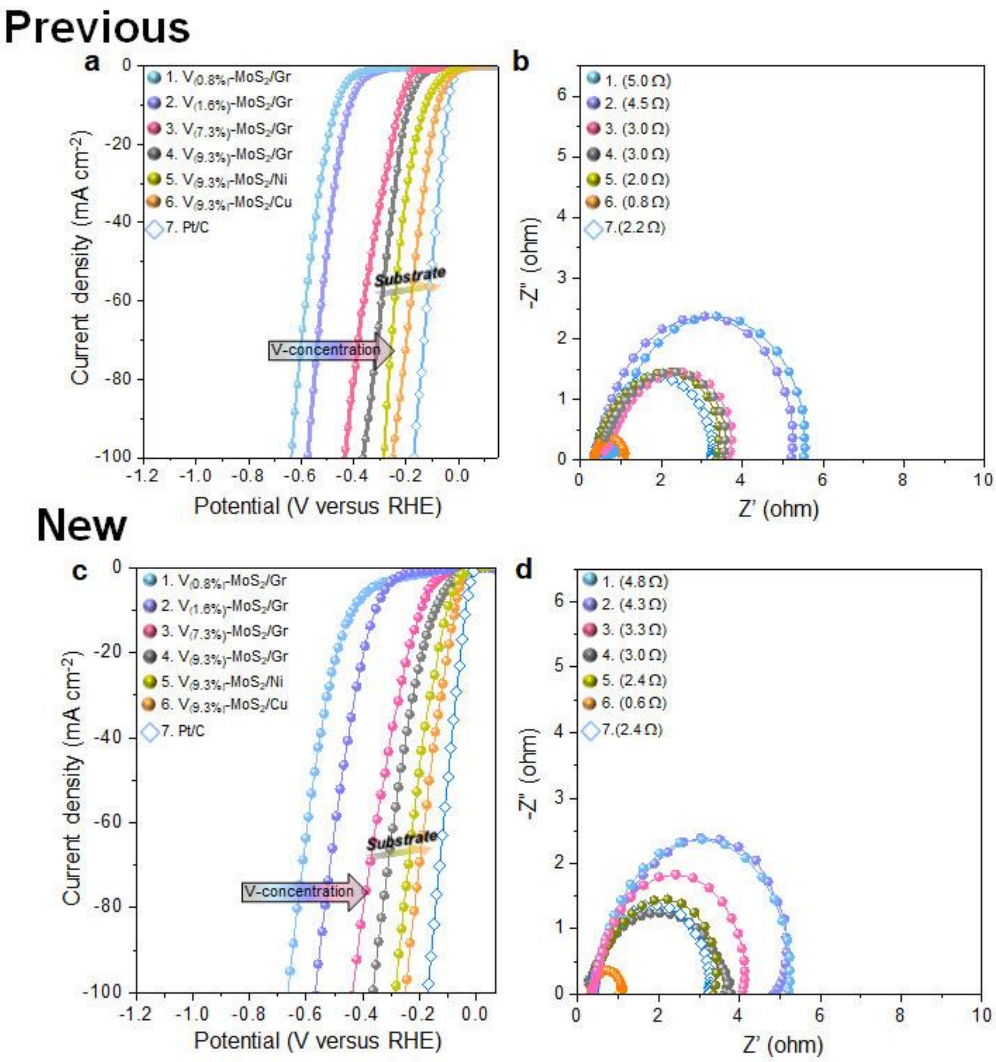

Figure S30. **Comparison of HER performance between previous and new V-MoS$_2$ samples**. Polarization curves and charge transfer resistance (R_{ct}) for (a-b) previous and (c-d) new V-MoS$_2$ samples. Both previous and new samples show similar HER performance.

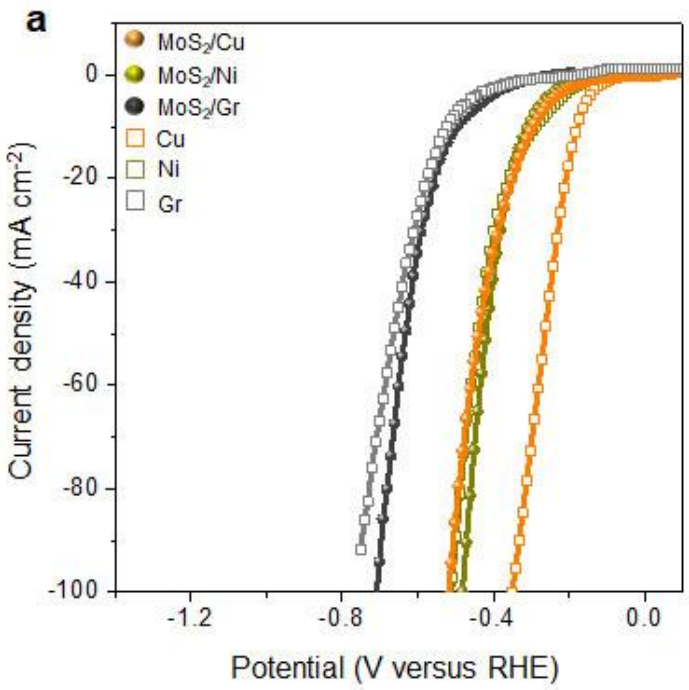

Figure S31. a,b) Polarization curves for pristine MoS_2 on Gr, Cu, and Ni substrates.

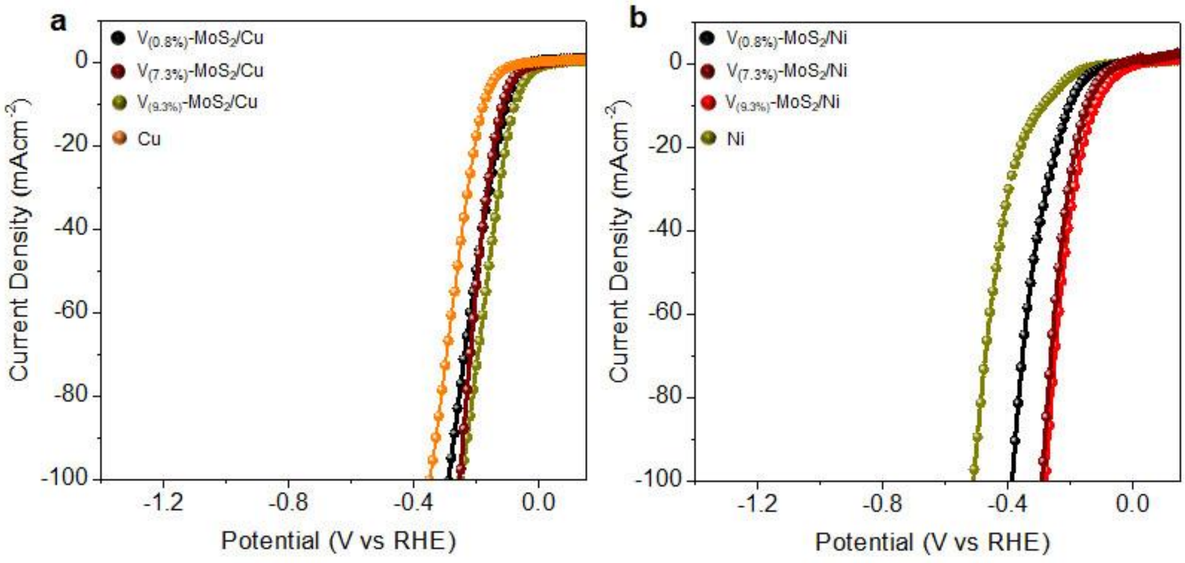

Figure S32. Polarization curves of V-MoS_2 on Cu and Ni substrates. a,b) Polarization curves for $V_{(0.8\%)}$-MoS_2, $V_{(7.3\%)}$-MoS_2, and $V_{(9.3\%)}$-MoS_2 on Cu and Ni substrates, respectively.

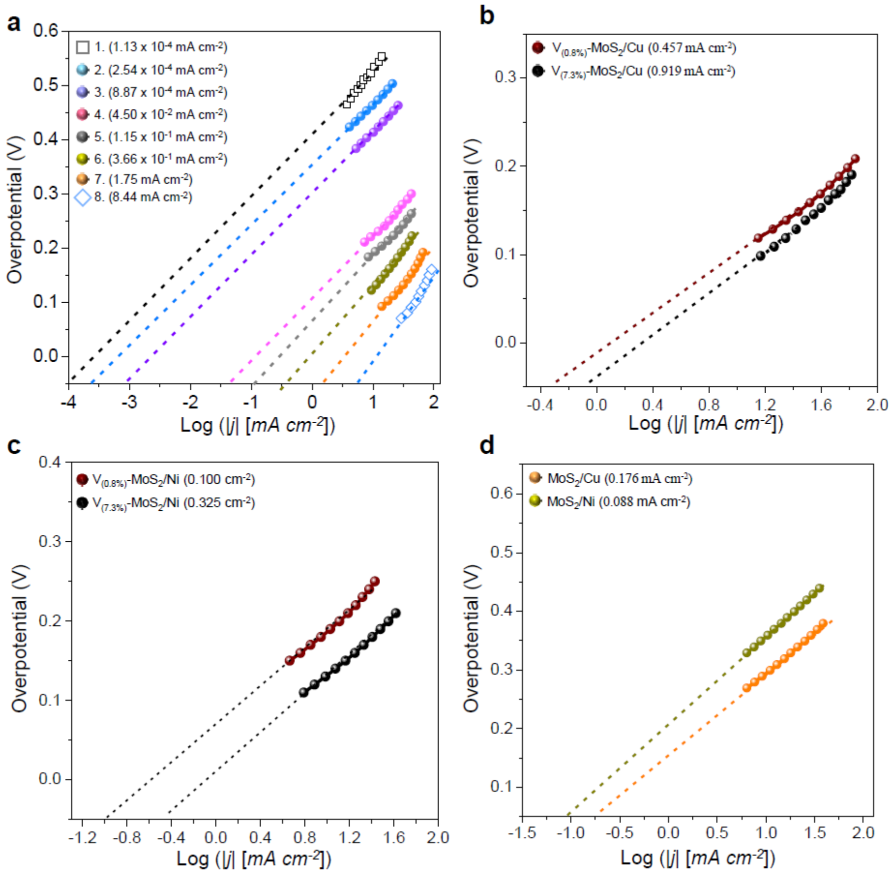

Figure S33. Exchange current density (j_0) determination by extrapolation. Estimated j_0 for a) pristine MoS_2 on graphite (Gr) substrate, $V_{(0.8\%)}$-MoS_2/Gr, $V_{(1.6\%)}$-MoS_2/Gr, $V_{(7.3\%)}$-MoS_2/Gr, $V_{(9.3\%)}$-MoS_2/Gr, $V_{(9.3\%)}$-MoS_2/Ni, $V_{(9.3\%)}$-MoS_2/Cu, Pt b-d) $V_{(0.8\%)}$-MoS_2, , $V_{(7.3\%)}$-MoS_2 and pristine MoS_2 on copper (Cu) and nickel (Ni) substrates.

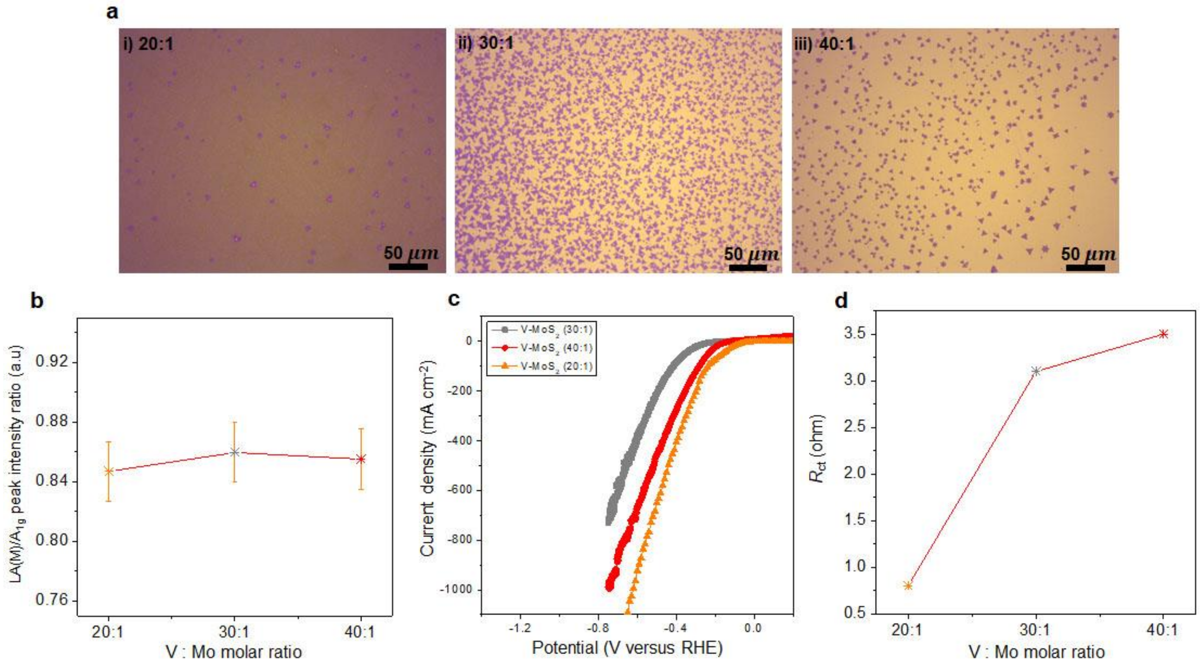

Figure S34. HER performance with higher V to Mo precursor molar ratio. a) Optical microscopy images of as-grown V-MoS_2 with higher molar ratio of V to Mo precursor of 20:1, 30:1 and 40:1, respectively. While the V-MoS_2 film was grown with the molar ratio of 20:1, only V-MoS_2 flakes were achieved with higher molar ratio of vanadium precursor. b) Raman intensity ratio of LA(M)/A_{1g} for as-grown V-MoS_2. c-d) Polarization and charge transfer resistance of V-MoS_2 on Cu substrate.

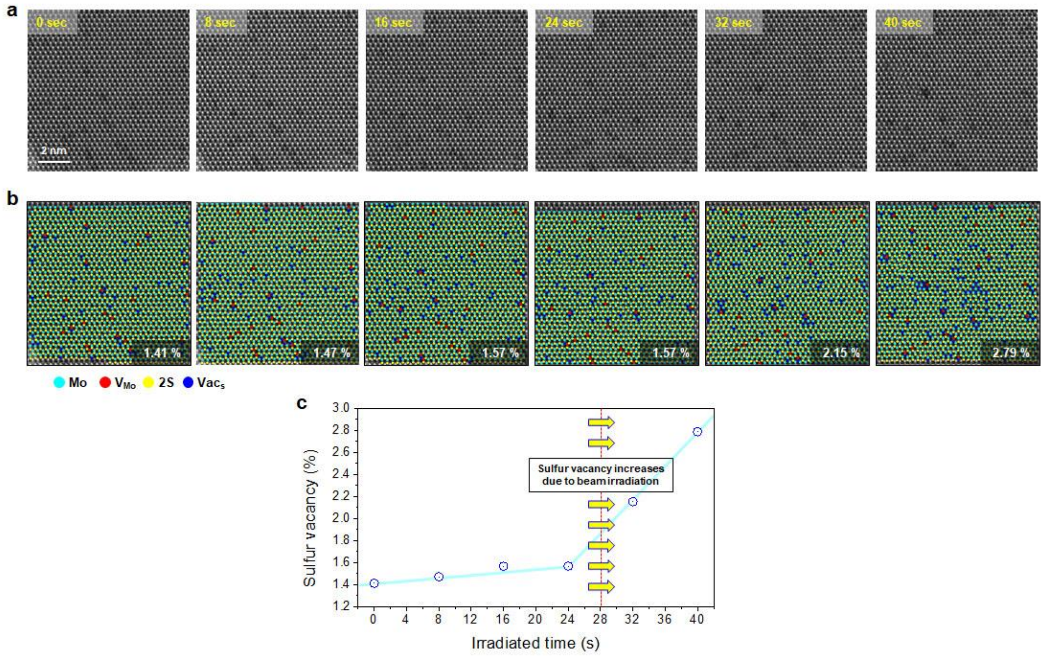

Figure S35. Time-dependent e-beam irradiation analysis using continuous electron beam scanning at 80 kV with a probe current of 25 pA. a) Continuously acquired ADF-STEM images and b) corresponding atomic maps. c) Statistics for S vacancy concentration with irradiation time in sec. In this work, ADF-STEM was acquired within 10 sec with a scanning rate of 8 μs/pixel for a 1024 × 1024 pixels resolution. Therefore, electron beam damage of the sample can be excluded.

Table S1. S vacancy (vac$_s$) formation energy of MoS_2 with secondary cation relative to that of pristine MoS_2. The value of 0.22 eV for V means that the formation of vac$_s$ next to a V atom is more stable than next to Mo atom.

Cation	ΔE (eV)
V	0.22
Nb	0.12
Ta	0.05

Table S2. Comparison of catalytic parameters for V-MoS_2 in terms of the V concentration and the substrate: onset potential, overpotential at 10 mA cm^{-2} (η_{10}), double layer capacitance (C_{dl}), relative electrochemically active surface area (EASA) extracted with respect to the substrate and charge transfer resistance (R_{ct}).

Sample	Onset Potential (mV)	η_{10} (mV)	C_{dl} (mF cm^{-2})	Relative EASA	$R_{ct}(\Omega)$
$V_{(0.8\%)}$-MoS_2/Gr	-332	-476	15.3	1.18	5
$V_{(1.6\%)}$-MoS_2/Gr	-254	-423	18.4	1.42	4.5
$V_{(7.3\%)}$-MoS_2/Gr	-127	-226	24.6	1.89	3.0
$V_{(9.3\%)}$-MoS_2/Gr	-72	-185	28.2	2.17	3.0
$V_{(9.3\%)}$-MoS_2/Ni	-50	-119	30.1	2.46	2.0
$V_{(9.3\%)}$-MoS_2/Cu	-30	-80	35.3	2.73	0.8
Pristine MoS_2/Cu	-228	-300	16.2	1.17	6.0
Pristine MoS_2/Ni	-245	-313	14.8	1.21	7.6
Pristine MoS_2/Gr	-343	-503	13.2	1.02	11.1
Bare Cu	-100	-209	13.8	1.00	5.0
Bare Ni	-150	-290	12.2	1.00	6.0
Bare Gr	-373	-524	13.0	1.00	9.8
Pt/C	-25	-41	-	-	2.2

Table S3. Comparison of onset potential (onset potential$_{normalized}$) and overpotential at 10 mA/cm^2 ($\eta_{10normalized}$) after EASA normalization.

Material	onset potential$_{normalized}$ (mV)	$\eta_{10normalized}$ (mV)	Reference
$V_{(9.3\%)}$-MoS_2	-30	-120	Our work
MoS_2	-53	-171	17
Co_2P@CP	-60	-140	18
Ni-W_2C	-50	-300	19
Ni-Pt film	-80	-250	20

Table S4. Comparison of catalytic parameters: Onset potential, overpotential at 10 (η_{10}) mA cm^{-2} and charge transfer resistance (R_{ct}) for previous and new V-MoS_2 samples. Similar values are recorded.

Sample	Onset Potential (mV)	η_{10} (mV)	R_{ct} (Ω)
$V_{(0.8\%)}$-MoS_2/Gr (Previous)	-332	-476	5
$V_{(1.6\%)}$-MoS_2/Gr (Previous)	-254	-423	4.5
$V_{(7.3\%)}$-MoS_2/Gr (Previous)	-127	-226	3.0
$V_{(9.3\%)}$-MoS_2/Gr (Previous)	-72	-185	3.0
$V_{(9.3\%)}$-MoS_2/Ni (Previous)	-50	-119	2.0
$V_{(9.3\%)}$-MoS_2/Cu (Previous)	-30	-80	0.8
Pt/C (Previous)	-25	-41	2.2
$V_{(0.8\%)}$-MoS_2/Gr (New)	-323	-476	5
$V_{(1.6\%)}$-MoS_2/Gr (New)	-253	-425	4.3
$V_{(7.3\%)}$-MoS_2/Gr (New)	-124	-220	3.0
$V_{(9.3\%)}$-MoS_2/Gr (New)	-81	-174	3.0
$V_{(9.3\%)}$-MoS_2/Ni (New)	-52	-109	2.4
$V_{(9.3\%)}$-MoS_2/Cu (New)	-30	-74	0.6
Pt/C (New)	-26	-42	2.4

Table S5. Exchange current density (j_0) contribution by vac_s in pristine and $V_{(9.3\%)}$-MoS_2.

Material	J_0 (mA cm^{-2})	% Mo-vac_s	% V-vac_s
Pristine MoS_2	0.176	2.1	0
$V_{(9.3\%)}$-MoS_2	1.75	0.8	1.7

References

[S1] Z. Lin, B. R. Carvalho, E. Kahn, R. Lv, R. Rao, H. Terrones, M. A. Pimenta, M. Terrones, *2D Mater*. **2016**, *3*, 022002.

[S2] E.Z. Xu, H. M. Liu, K. Park, Z. Li, Y. Losovyj, M. Starr, M. Werbianskyj, H. A. Fertig, S. X. Zhang, *Nanoscale* **2019**, *9*, 3576.

[S3] K. Karthick, T. K. Bijoy, A. Sivakumaran, A. B. M. Basha, P. Murugan, S. Kundu, *Inorg. Chem.* **2020**, *59*, 10197.

[S4] Y. Liao, *Practical Electron Microscopy and Database,* Global Sino, **2006**.

[S5] A. W. Robertson, Y. Lin, S. Wang, H. Sawada, C. S. Allen, Q. Chen, S. Lee, G. Lee, J. Lee, S. Han, E. Yoon, A. I. Kirkland, H. Kim, K. Suenaga, J. H. Warner, *ACS Nano* **2016**, *10*, 10227.

[S6] Y. Lin, S. Li, H. Komsa, L. Chang, A. V. Krasheninnikov, G. Eda, K. Suenaga, *Adv. Funct. Mater.* **2018**, *28,* 1704210.

[S7] S. Park, S. J. Yun, Y. I. Kim, J. H. Kim, Y. -M. Kim, K. K. Kim, Y. H. Lee, *ACS Nano* 2020, *14*, 8784.

[S8] J. Yang, A. R. Mohmad, Y. Wang, R. Fullon, X. Song, F. Zhao, I. Bozkurt, M. Augustin, E. J. G. Santos, H. S. Shin, W. Zhang, D. Voiry, H. Y. Jeong, M. Chhowalla, *Nat. Mater.* **2019**, *18*, 1309.

[S9] R. He, J. Hua, A. Zhang, C. Wang, J. Peng, W. Chen, J. Zeng, *Nano Lett.* **2017**, *17*, 4311.

[S10] G. Li, D. Zhang, Q. Qiao, Y. Yu, D. Peterson, A. Zafar, R. Kumar, S. Curtarolo, F. Hunte, S. Shannon, Y. Zhu, W. Yang, L. Cao, *J. Am. Chem. Soc.* **2016**, *138*, 16632.

[S11] T.F. Jaramillo, K. P. Jorgensen, J. Bonde, J. H. Nielsen, S. Horch, Chorkendorff, *Science* **2007**, *317*, 100.

[S12] D. Vikraman, S. Hussain, K. Akbar, L. Truong, A. Kathalingam, S. H. Chun, J. Jung, H. J. Park, H. S. Kim, *ACS Sustain. Chem. Eng*. **2018**, *6*, 8400.

[S13] D. Voiry, H. Yamaguchi, J. Li, D. C. Alves, T. Fujita, M. Chen, T, Asefa, V. B. Shenov, G. Eda, M. Chhowalla, *Nat. Mater.* **2013**, *12*, 850.

[S14] X. Fan, Y. Liu, Z. Peng, Z. Zhang, H. Zhou, X. Zhang, B. I. Yakobson, W. A. Goddard, X. Guo, R. H. Hauge, J. M. Tour, *ACS Nano* **2017**, *11*, 384.

[S15] Y. Yu, G. H. Nam, Q. He, X. J. Wu, K. Zhang, Z. Yang, J. Chen, Q. Ma, M. Zhao, Z. Liu, F. R. Ran, X. Wang, H. Li, X. Huang, B. Li, Q. Xiong, Q. Zhang, Z. Liu, L. Gu, Y. Du, W. Huang, H. Zhang, *Nat. Chem*. **2018**, *10*, 638.

[S16] H. Wang, Z. Lu, S. Xu. D. Kong, J. J. Cha, G. Zheng, P. C. Hsu, K. Yan, D. Bradshaw, F. B. Prinz, Y. Cui *PNAS* **2013**, *110*, 19701.

[S17] X. Kong, X. Shen, C. Zhang, S. N. Oliaee, Z. Peng *Inorg. Chem. Front.* **2016**, *3*, 1376.

[S18] Y. Zhang, L. Gao, E. J. Hensen, J. P. Hofmann *ACS Energy Lett.* **2018**, *3*, 1360.

[S19] E. H. Ang, K. N. Dinh, X. Sun, Y. Huang, J. Yang, Z. Dong, X. Dong, W. Huang, Z. Wang, H. Zhang, Q. Yan *AAAS Res.* **2019**, *2019*, 4029516.

[S20] K. Eiler, S. Surinach, J. Sort, E. Pellicer *Appl. Catal. B. Environ*. **2020**, *265*, 118597.
